# The cell adhesion protein dystroglycan affects the structural remodeling of dendritic spines

**DOI:** 10.1038/s41598-022-06462-7

**Published:** 2022-02-15

**Authors:** Izabela Figiel, Ewa Bączyńska, Tomasz Wójtowicz, Marta Magnowska, Anna Buszka, Monika Bijata, Jakub Włodarczyk

**Affiliations:** 1 Laboratory of Cell Biophysics, Nencki Institute of Experimental Biology, Polish Academy of Sciences, Warsaw, Poland; 2 Laboratory of Charge Transfer Processes in Hydrodynamic Systems, Institute of Physical Chemistry, Polish Academy of Sciences, Warsaw, Poland

**Keywords:** Cell biology, Neuroscience

## Abstract

Dystroglycan (DG) is a cell membrane protein that binds to the extracellular matrix in various mammalian tissues. The function of DG has been well defined in embryonic development as well as in the proper migration of differentiated neuroblasts in the central nervous system (CNS). Although DG is known to be a target for matrix metalloproteinase-9 (MMP-9), cleaved in response to enhanced synaptic activity, the role of DG in the structural remodeling of dendritic spines is still unknown. Here, we report for the first time that the deletion of DG in rat hippocampal cell cultures causes pronounced changes in the density and morphology of dendritic spines. Furthermore, we noted a decrease in laminin, one of the major extracellular partners of DG. We have also observed that the lack of DG evokes alterations in the morphological complexity of astrocytes accompanied by a decrease in the level of aquaporin 4 (AQP4), a protein located within astrocyte endfeet surrounding neuronal dendrites and synapses. Regardless of all of these changes, we did not observe any effect of DG silencing on either excitatory or inhibitory synaptic transmission. Likewise, the knockdown of DG had no effect on Psd-95 protein expression. Our results indicate that DG is involved in dendritic spine remodeling that is not functionally reflected. This may suggest the existence of unknown mechanisms that maintain proper synaptic signaling despite impaired structure of dendritic spines. Presumably, astrocytes are involved in these processes.

## Introduction

Dystroglycan (DG) is a member of the dystrophin-associated glycoprotein complex, which was first isolated from skeletal muscle^1^ but is widely expressed in many tissues. DG is encoded by a single gene (*DAG1*) and is cleaved into two proteins, α-DG and β-DG, by post-translational processing^2^. The DG complex provides an important structural link between the extracellular matrix and the intracellular actin cytoskeleton. The extracellular α-DG is highly glycosylated and binds to numerous laminin G domain ligands, including laminins, agrin, and perlecan^3,4^, and presynaptic neurexins in the brain^5^. The transmembrane β-DG anchors α-DG to the cell membrane via its N-terminal domain and interacts with the cytoskeletal proteins dystrophin and utrophin via its C-terminal cytoplasmic domain^6^. The hypoglycosylation of α-DG and the consequent lack of ligand-binding activity result in progressive muscular dystrophy often associated with brain deformities and intellectual disability^7^.

The exact role of the DG complex within the CNS is still not fully understood. Two decades ago, an elegant study by Zaccaria and colleagues^8^ on the adult mouse brain showed that DG is present in neurons of the cerebral cortex, hippocampus, olfactory bulb, basal ganglia, thalamus, hypothalamus, brainstem and cerebellum. They also found DG in astrocytes and endothelial cells at the blood–brain barrier. Tissue selective gene deletion enabled the recognition of the distinct functions of glial and neuronal DG in the brain^9^. These studies indicated that the extracellular interactions of α-DG expressed on glial cells stabilize glial limitans and scaffolding for the migration of neurons during forebrain development. On the other hand, DG expressed on neurons is required for normal hippocampal long-term potentiation. Notably, DG has been found on inhibitory GABAergic synapses and used as a GABAergic synapse marker^10,11^. Moreover, the expression of glycosylated α-DG is linked to the neuronal activity level and is essential for the homeostatic scaling up of GABAergic synaptic strength by regulating GABAA receptor abundance at the synapse^12^. Interestingly, DG was shown to be a substrate of MMP-9, which has only been identified at excitatory synapses so far^13^. Although the involvement of MMP-9 in synapse formation and stabilization has been repeatedly shown (reviewed in^14^), the role of the MMP-9-dependent proteolytic cleavage of DG in synaptic remodeling has not been determined.

Studies during the past few years have indicated that astrocytes are involved in the proper functioning of excitatory synapses^15^. These cells are characterized by high morphological complexity with extended and branched processes that are closely associated with synapses both at the structural level by covering dendritic spines and presynaptic terminals, and at the functional level through the release of gliotransmitters^16–19^. A particularly important characteristic of astrocytes is their ability to rapidly rearrange their processes and modify their coverage of the synaptic elements^20^. It is likely that deficits in the physical contacts between astrocytes and synapses contribute to homeostatic dysfunction in neurological diseases. Recent reviews have provided insight into the cellular and molecular mechanisms underlying dynamic remodeling in astrocyte morphology during injury and the development of pathology^21,22^. In the hippocampal CA1 region of adult rats, perisynaptic astrocytic processes are present in approximately 62% of synapses^23^. Similarly, in the CA1 area of organotypic hippocampal slice cultures, more than 85% of spine synapses are also in contact with glial protrusions^24^. Since the glia-specific deletion of DG in the mouse brain resulted in the disorganization of the astroglial endfeet structures^25^, it is reasonable to speculate that astrocyte-derived DG may be involved in synaptic formation and function.

We have previously demonstrated that DG plays a major role in the dendritic morphogenesis of hippocampal neurons during early stages of their in vitro differentiation^26^. Here, we investigated whether DG affects the dendritic tree arborization and structural remodeling of dendritic spines in mature cultures. Using a lentiviral vector encoding a DG-targeted shRNA, we examined the effect of DG silencing on the complexity of dendritic trees as well as the shape and density of dendritic spines. We also studied the influence of DG knockdown on the morphology of astrocytes present in the culture. In addition, we investigated basal excitatory and inhibitory synaptic transmission and the levels of Psd-95, a protein crucial for synaptic plasticity, in DG-deficient hippocampal cultures. Our results show that the loss of DG causes changes in the complexity of dendritic arbors as well as in the shape and density of neuronal dendritic spines together with alterations in astrocyte morphology. These changes were accompanied by lower expression of laminin and AQP4, known ligands of DG. Interestingly, these structural changes neither affected the basal synaptic transmission nor the level of the Psd-95 protein. Overall, our results indicate that altered spine morphology in DG-deficient hippocampal neurons does not lead to synaptic dysfunction. Therefore, comprehensive research is essential for a more complete understanding of this phenomenon. It seems appropriate to consider the contribution of neural-glial interactions in maintaining normal synaptic transmission.

## Results

### Knockdown of dystroglycan influences dendritic spine shape and density

**Figure 1:**
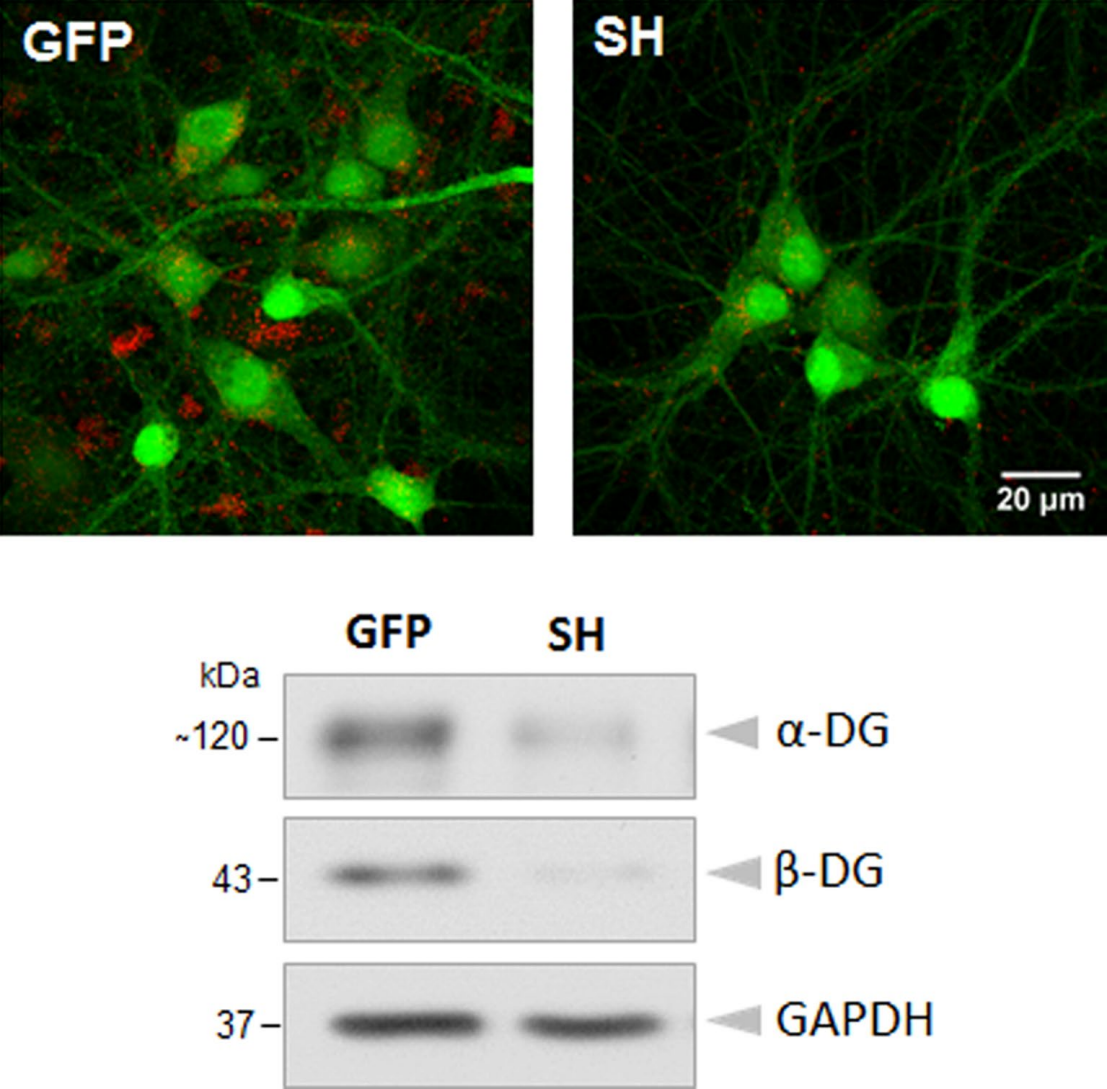
The efficiency of dystroglycan silencing in the hippocampal cell cultures. Hippocampal cells were infected on the 9th day after seeding with a lentivirus carrying shRNA for DG (SH) or with an empty lentivirus carrying only GFP (GFP). After 11 days, the cultures were subjected to immunocytochemical staining and western blot analysis. Upper panel: Confocal images of the hippocampal cultures infected with the given viruses and subjected to immunofluorescence staining with a β-DG-specific antibody (red). Lower panel: Western blots showing a decrease in the α- and β-DG levels in the cultures infected with virus carrying shRNA targeting DG (SH) compared to those in the control cultures (GFP). GAPDH served as a loading control. Original blots are presented in Supplementary Fig. S6.

To study the role of DG in neuronal structure and function, we used a lentiviral vector to deliver shRNA specifically targeting DG into cultured hippocampal cells. Infection with this virus effectively blocked DG expression in both cell types, namely, in neurons and astrocytes, as revealed by the immunocytochemical staining results and western blot analysis (Fig. 1 and Supplementary Figs. S1 and S6). We have previously reported that reduction of DG expression disrupts dendrite growth and arborization in developing hippocampal neurons^26^. To determine whether the deletion of DG influences the complexity of dendritic arbors in mature neurons, on day 20 in vitro, we performed imaging of cells infected with either the lentivirus carrying shRNA targeting DG (SH) or an empty virus encoding green fluorescent protein (GFP), and additionally transfected with a red fluorescent protein (RFP)-encoding vector. The Sholl analysis of neurons upon DG knockdown revealed a downward shift of the plot compared with the GFP control, indicating a decrease in the complexity of dendritic trees. Interestingly, the total dendritic length did not change after DG silencing (Supplementary Fig. S3). These results indicate that DG is generally involved in the mechanisms controlling dendritic branching.

**Figure 2:**
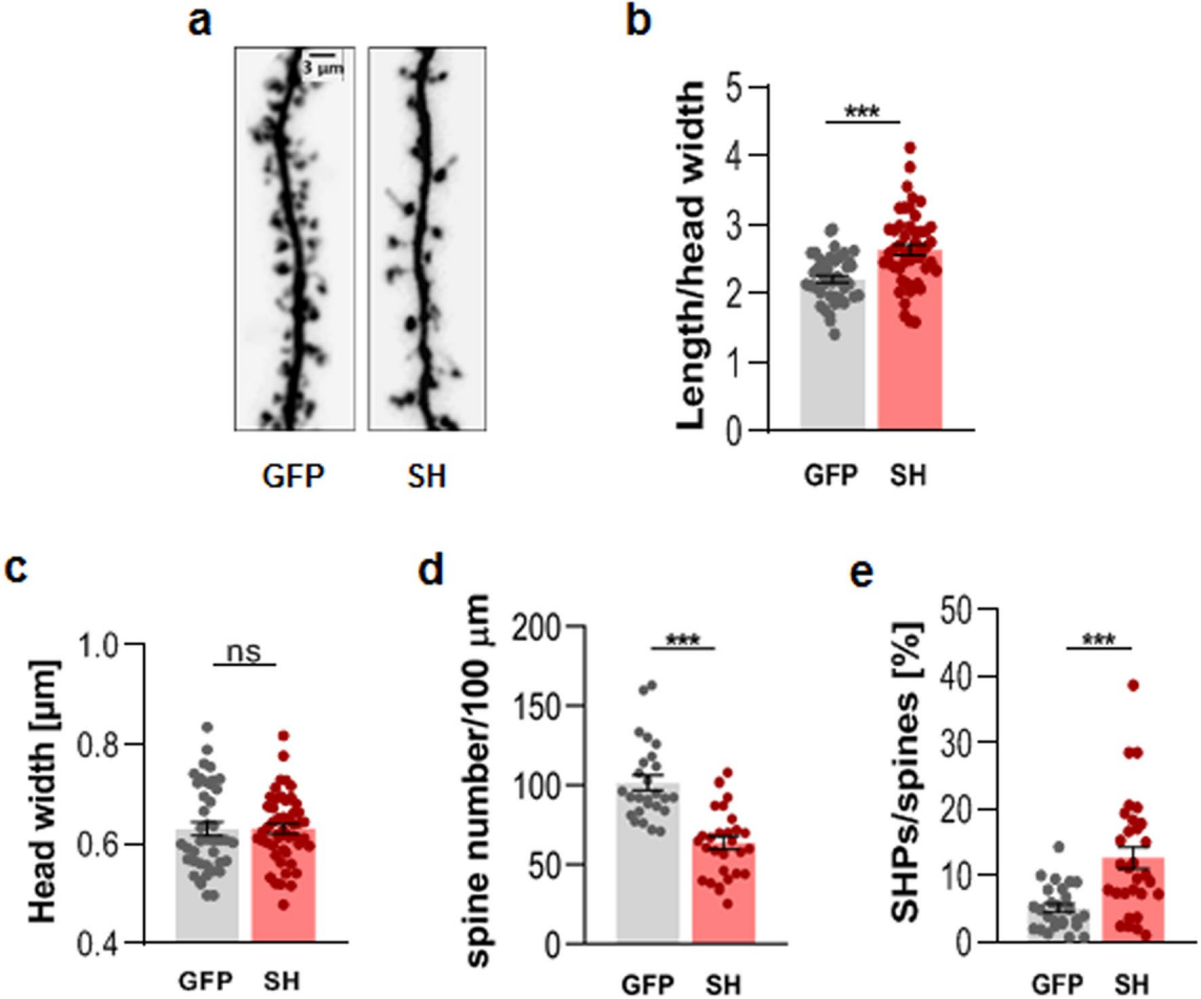
Knockdown of dystroglycan in cultured hippocampal cells induces changes in dendritic spine shape and density. (**a**) Representative images of the dendrites of neurons infected with either an empty lentivirus (GFP) or a lentivirus carrying shRNA for DG (SH). (**b**) The bar plot shows a significant increase in the length/head width ratio of the dendritic spines of neurons with silenced DG expression. (**c**) The bar plot shows no difference in the spine head width, (**d**) The bar plot shows the significantly reduced density of dendritic spines (defined as the number of spines per 100 µm of dendrite) in the neurons with reduced DG expression (SH) compared to that in the control neurons (GFP). (**e**) The bar plot shows the quantification of spine head protrusions (SHPs). The knockdown of DG results in an increase in the percentage of SHPs. The results are expressed as the mean ± SEM, ***p < 0.001.

To evaluate the number and shape of dendritic spines, we performed a morphometric analysis of pyramidal neurons from cultures infected with aforementioned viruses and transfected with RFP-encoding vector (Fig. 2a). The results showed a significant increase in the spine length/head width ratio (GFP: 2.22 ± 0.05, n = 3011 spines, N = 26 cells; SH: 2.64 ± 0.08, n = 2791 spines, N = 30 cells; *p* < 0.001; Fig. 2b). Interestingly, DG silencing affected only the spine length, as the head width did not change (*p *= 0.68; Fig. 2c). We also observed a reduction in spine density in neurons silenced for DG (GFP: 101.50 ± 4.83 spines/100 μm, N = 26 cells, analyzed dendritic length: 15414.1 μm; SH: 63.69 ± 4.05 spines/100 μm, N = 29 cells, analyzed dendritic length: 15018.6 μm; *p* < 0.001; Fig. 2d). Additionally, these neurons were characterized by a marked increase in the percentage of filopodia-like processes emerging from the dendritic spine head, which are called spine head protrusions (SHPs) (GFP: 5.165% ± 0.66%, N = 26 cells; SH: 12.7% ± 1.61%, N = 30 cells; *p* < 0.001; Fig. 2e). These results indicate that DG knockdown in primary hippocampal cultures causes significant changes in the morphology and density of dendritic spines.

**Figure 3:**
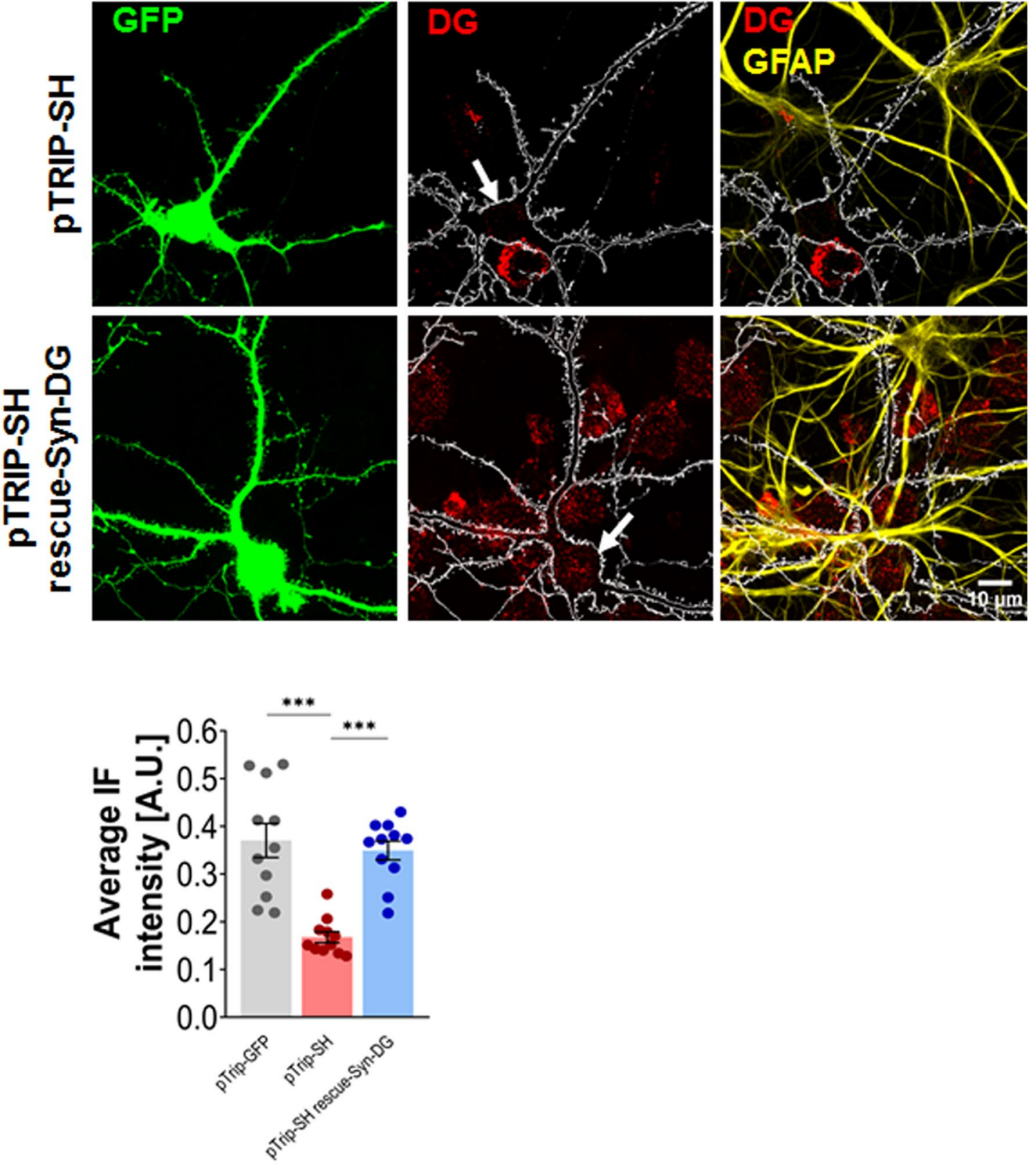
Co-transfection of hippocampal cultures with pTrip-SH and pTrip rescue-Syn-DG vectors results in the re-expression of neuronal DG at endogenous levels. Primary hippocampal cultures were transfected with the given plasmids on the 9th day after seeding. Following 11 days, the cultures were subjected to immunocytochemical staining with anti-β-DG (red) and anti-GFAP (yellow) antibodies. Upper panel: Confocal images showing the results of immunostaining. The arrows indicate transfected cells. Lower panel: The effect of DG knockdown and re-expression was estimated based on the average intensity of the β-DG immunofluorescence (IF) signal in transfected cells. The results are expressed as the mean ± SEM, ***p < 0.001.

**Figure 4:**
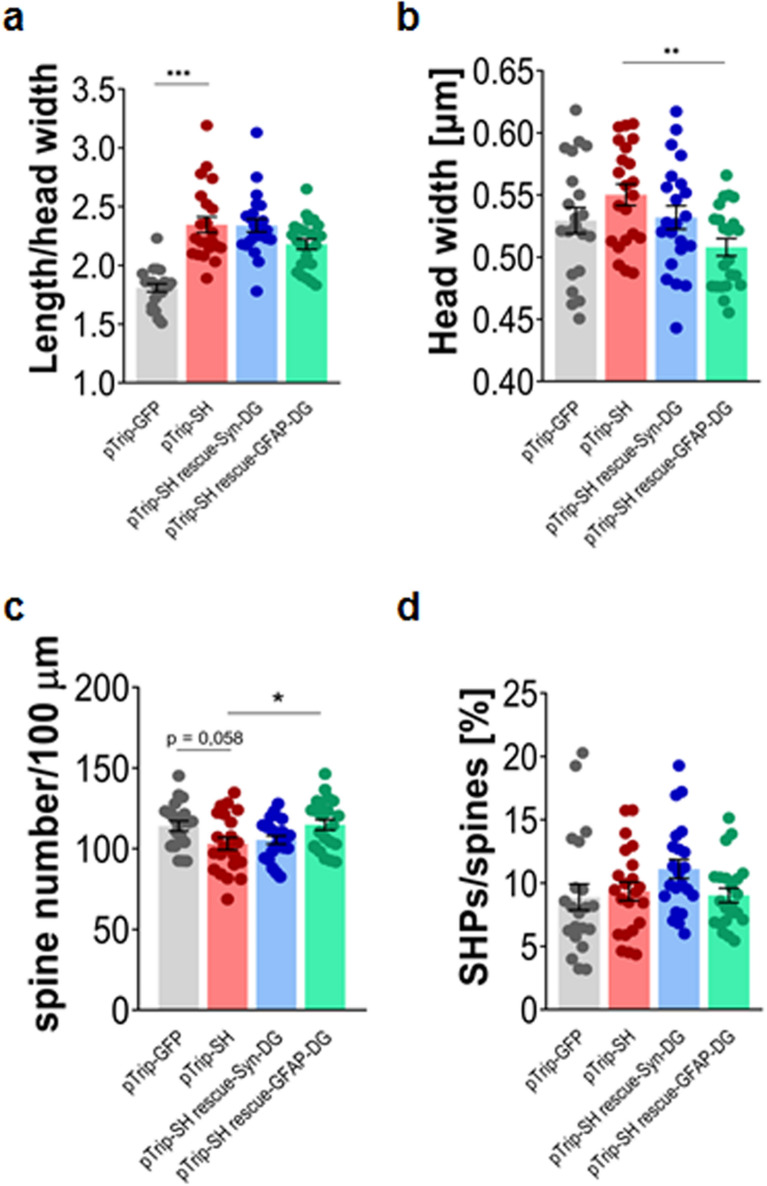
The effect of DG silencing is partially rescued by astroglial overexpression of an shRNA-resistant DG. Primary hippocampal cultures were co-transfected with pTrip-GFP, pTrip-SH, pTrip-SH-rescue-Syn-DG or pTrip-SH-rescue-GFAP-DG. Morphometric analysis of dendritic spines was performed at 20 DIV. (**a**) The bar plot shows a significant increase in the length/head width ratio of the dendritic spines of pTrip-SH-transfected neurons compared with control (pTrip-GFP). Neither the overexpression of DG in neurons nor in astrocytes reversed this phenotype. (**b**) The bar plot shows significant decrease in the spine head width of neurons from cultures transfected with pTrip-SH-rescue-GFAP-DG. (**c**) The bar plot shows the significantly increased density of dendritic spines in neurons from cultures transfected with pTrip-SH-rescue-GFAP-DG compared to neurons from cultures transfected with pTrip-SH. (**d**) The bar plot shows the quantification of spine head protrusions (SHPs). No differences were found between the cultures transfected with the given vectors. The results are expressed as the mean ± SEM, *p = 0.03; **p = 0.003.

To confirm that the observed remodeling of dendritic spines was dependent on the DG expression in neurons, we performed rescue experiments. We created a DNA construct, under the synapsin promoter, in which silent mutation was introduced into the cDNA coding region for DG rescue (rescue-Syn-DG), which was transcribed into mRNA that could not be recognized by shRNA. Although the co-expression of rescue-Syn-DG with pTrip-SH in neurons resulted in the re-expression of DG at endogenous levels (Fig. 3), a reversal of the DG knockdown-induced phenotype was not achieved. Overexpression of DG in neurons neither affected the length-to-width ratio parameter, which was caused by DG knockdown (pTrip-SH: 2.35 ± 0.07, n = 2980 spines, N = 22 cells; pTrip-SH rescue-Syn-DG: 2.34 ± 0.05, n = 3765 spines, N = 22 cells; *p* > 0.99; Fig. 4a) nor the spine head width (pTrip-SH; 0.55 ± 0.01 μm; pTrip-SH rescue-Syn-DG: 0.53 ± 0.01 μm; *p* = 0.38; Fig. 4b). We also noted no changes in spine density (pTrip-SH: 103.10 ± 3.81 spines/100 μm, N = 22 cells, analyzed dendritic length: 4574.84 μm; pTrip-SH rescue-Syn-DG: 105.40 ± 2.60 spines/100 μm, N = 22 cells, analyzed dendritic length: 4626.77 μm; *p* = 0.94; Fig. 4c) as well as in the percentage of SHPs (pTrip-SH: 9.35% ± 0.73%, N = 22 cells; pTrip-SH rescue-Syn-DG: 11.11% ± 0.76%, N = 22 cells; *p* = 0.38; Fig. 4d). Interestingly, rescue of DG expression specifically in astrocytes (rescue-GFAP-DG) led to an increase in spine density, corresponded to that observed in neurons transfected with control plasmid, and abolished the DG silencing effect (pTrip-SH: 103.10 ± 3.81 spines/100 μm, N = 22 cells, analyzed dendritic length: 4574.84 μm; pTrip-SH rescue-GFAP-DG: 114.80 ± 3.29 spines/100 μm, N = 22 cells, analyzed dendritic length: 4781.66 μm; *p* = 0.03; Fig. 4c). Additionally, we observed a decrease in the spine head width after transfection with pTrip-SH rescue-GFAP-DG vector compared to cells transfected with pTrip-SH plasmid alone (pTrip-SH rescue-GFAP-DG: 0.51 ± 0.01 μm; pTrip-SH: 0.55 ± 0.01 μm; *p* = 0.003; Fig. 4b). These data indicate that DG shRNA-induced changes in the number of dendritic spines resulted from the specific knockdown of DG rather than from off-target effects.

### Overexpression of dystroglycan in hippocampal neurons does not affect the structure of dendritic spines

**Figure 5:**
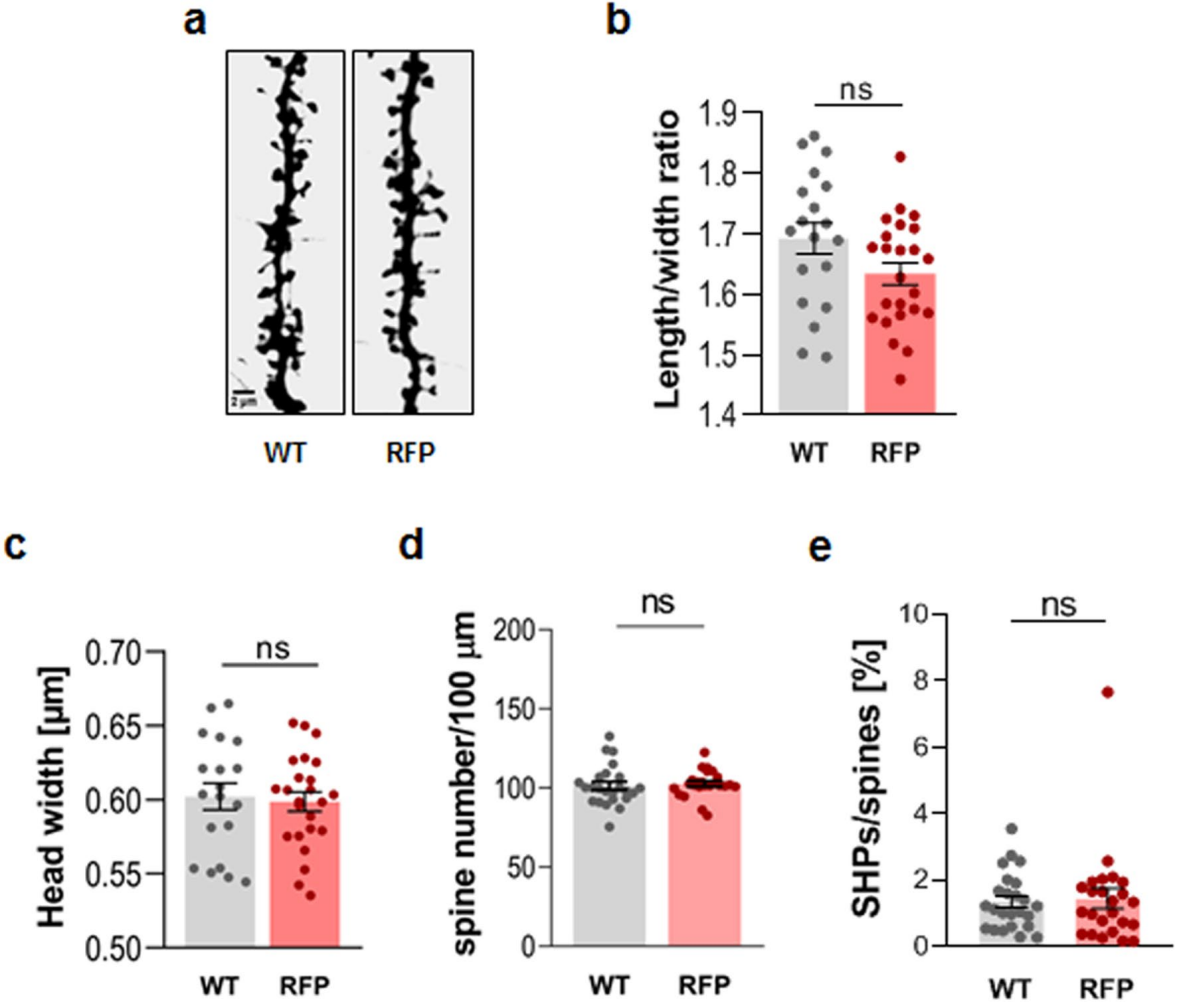
Overexpression of dystroglycan in hippocampal neurons does not affect the shape of dendritic spines. (**a**) Representative images of the dendrites of neurons transfected with a plasmid encoding DG (WT) or a control plasmid (RFP). The bar plots show the results of the morphometric analysis of the dendritic spines. Overexpression of DG in neurons did not change any of the parameters tested, namely: (**b**) length/head width ratio, (**c**) head width, (**d**) spine density, and (**e**) percentage of SHPs. The results are expressed as the mean ± SEM.

Substantial changes in the morphology and density of dendritic spines were observed after the disruption of DG expression in both neurons and astrocytes present in the hippocampal cell culture. Therefore, in the next experiment, we attempted to determine whether the overexpression of DG exclusively in neurons causes structural changes in dendritic spines. For this purpose, the cultures were transfected with a plasmid encoding DG (WT) under the control of the synapsin promoter. The effectiveness of this construct was confirmed in our previous studies^26^. For better visualization of the cellular morphology, a plasmid encoding RFP was co-transfected into the neurons with the DG overexpression vector (Fig. 5a). The morphometric analysis of dendritic spines showed a lack of significant differences in spine shape between neurons expressing WT DG (WT: 1.69 ± 0.026, n = 2334 spines, N = 19 cells) and control neurons transfected only with the RFP vector (RFP: 1.46 ± 0.018, n = 2688, N = 24 cells) (p = 0.06; Fig. 5b). We did not observe any changes in the spine head width (p = 0.74; Fig. 5c). Likewise, DG overexpression had no effect on spine density (p = 0.56; Fig. 5d) and the percentage of SHPs (p = 0.42; Fig. 5e). Thus, the enhanced neuronal expression of DG did not affect the structure of dendritic spines. These results, along with the results obtained from the rescue experiments, may suggest that astrocytes and the DG located in the astrocytic protrusions surrounding the synapses have a role in the remodeling of dendritic spines.

### Knockdown of dystroglycan affects aquaporin-4 and laminin expression

**Figure 6:**
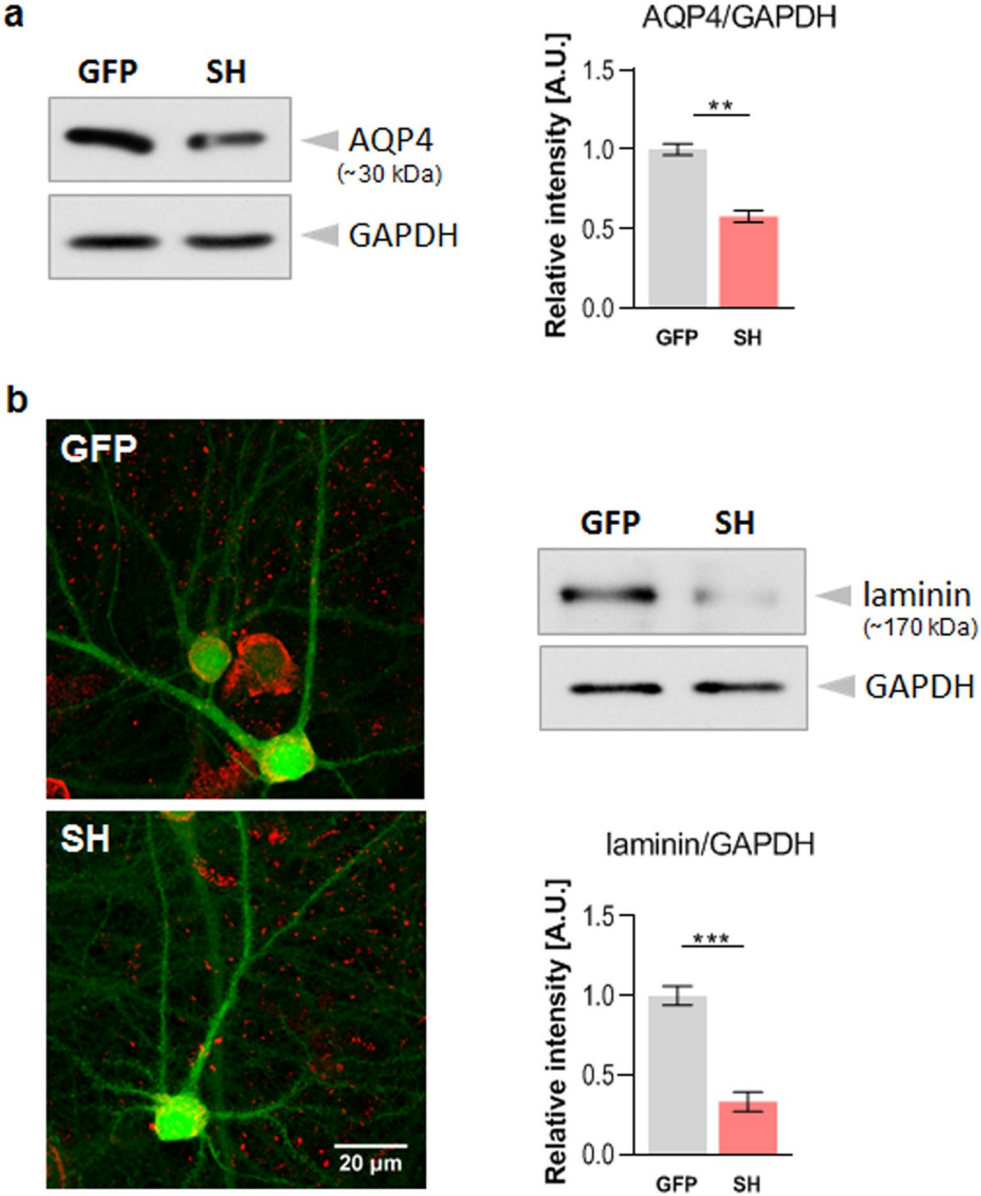
Knockdown of dystroglycan reduces the expression of AQP4 and laminin. (**a**) Left panel: Representative western blot (of three independent experiments) showing the AQP4 protein levels in the lysates from hippocampal cell cultures infected with a virus carrying shRNA against DG (SH) or a virus carrying the GFP gene (GFP). GAPDH was used as a loading control. Original blots are presented in Supplementary Fig. S7. Right panel: Bar graph showing the densitometric analysis of the blots probed for AQP4 and GAPDH. (**b**) Left panel: Confocal immunofluorescence images showing laminin expression (red) in hippocampal cell cultures infected with a virus carrying shRNA against DG (SH) or an empty virus (GFP). Right panel: Representative western blot (of four independent experiments) showing the laminin levels in cell lysates from virus-infected cultures. GAPDH was used as a loading control. Original blots are presented in Supplementary Fig. S8. Bar graph shows the densitometric analysis of the blots probed for laminin and GAPDH. The results are expressed as the mean ± SEM. **p < 0.01, ***p < 0.001.

It is well known that DG located in astrocytes plays a key role in anchoring aquaporin-4 (AQP4), an astrocyte-specific water channel protein enriched in perivascular endfeet^27^ as well as at the ends of astrocytic processes associated with excitatory synapses^28,29^. Therefore, we examined whether the silencing of DG influences the expression level of AQP4 in hippocampal cultures. Protein extracts from cells infected with either the lentivirus containing the shRNA for DG (SH) or the empty lentivirus (GFP) were prepared 10 days after infection. The western blot analysis showed a clear decrease in the AQP4 levels in cells with reduced DG expression compared to those in the control cultures (*p* < 0.01; Fig. 6a). This reduction of AQP4 after DG suppression was not related to a decrease in the number of astrocytes as glial fibrillary acidic protein (GFAP) levels remained unchanged (Supplementary Fig. S4).

The cell-surface expression of AQP4 has been shown to be enhanced by laminin^30^, one of the main components of the extracellular matrix and key regulator of cell adhesion, migration and differentiation^4^. We investigated how DG silencing affects laminin expression in our in vitro model. First, the cultures infected with the given viruses were subjected to immunocytochemical staining with an antibody specific for laminin. The microscopic analysis of the obtained specimens revealed a marked decrease in laminin immunoreactivity in the cultures with reduced DG expression (SH) compared to that in the control (GFP) cultures (Fig. 6b). Likewise, the western blot analysis of the laminin levels in the protein extracts showed a clear decrease in the level of this protein after DG knockdown (*p* < 0.001; Fig. 6b). These results may indicate that DG silencing disrupts the interactions between cells and extracellular matrix, and this presumably could lead to changes in dendritic spine structure.

### Knockdown of dystroglycan leads to morphological changes in astrocytes

**Figure 7:**
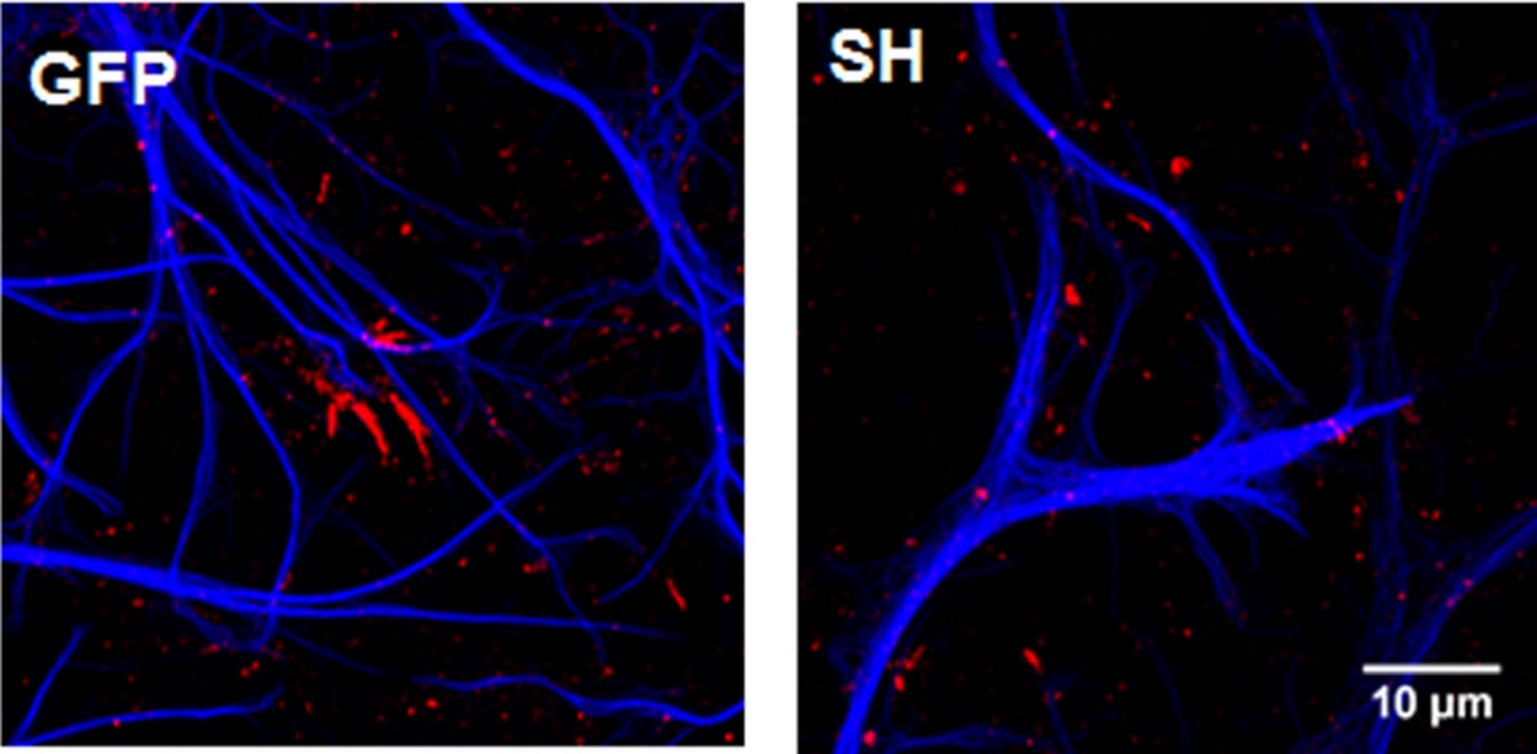
Knockdown of dystroglycan induces changes in astrocyte morphology. Confocal images of hippocampal cell cultures infected with a virus carrying shRNA against DG (SH) or an empty virus (GFP) immunostained for GFAP (blue) and pERM (red).

The AQP4-mediated movement of water across the plasma membrane is considered to be one of the possible mechanisms by which astrocytes adjust their volume and regulate the mobility of their processes near the synapses^31^. Therefore, the disruption in AQP4 expression accompanying DG silencing in hippocampal cultures should induce changes in astrocyte morphology. To investigate this possibility, lentivirus-infected cultures were subjected to double immunostaining of GFAP and phosphorylated ezrin/radixin/moesin (pERM). It has been established that phospho-ezrin (pEzrin) is involved in the formation of perisynaptic astrocytic processes and can be used as a marker of these protrusions, which are difficult to visualize as they are smaller than the resolution of a light microscope^32^. The astrocytes from the control cultures displayed a fingerlike pattern of pERM immunostaining, whereas the astrocytes with reduced DG expression showed discrete pERM-positive puncta (Fig. 7). These results indicate that the knockdown of DG leads to the retraction of astrocytic processes.

### Knockdown of dystroglycan does not affect either excitatory or inhibitory synaptic transmission

**Figure 8:**
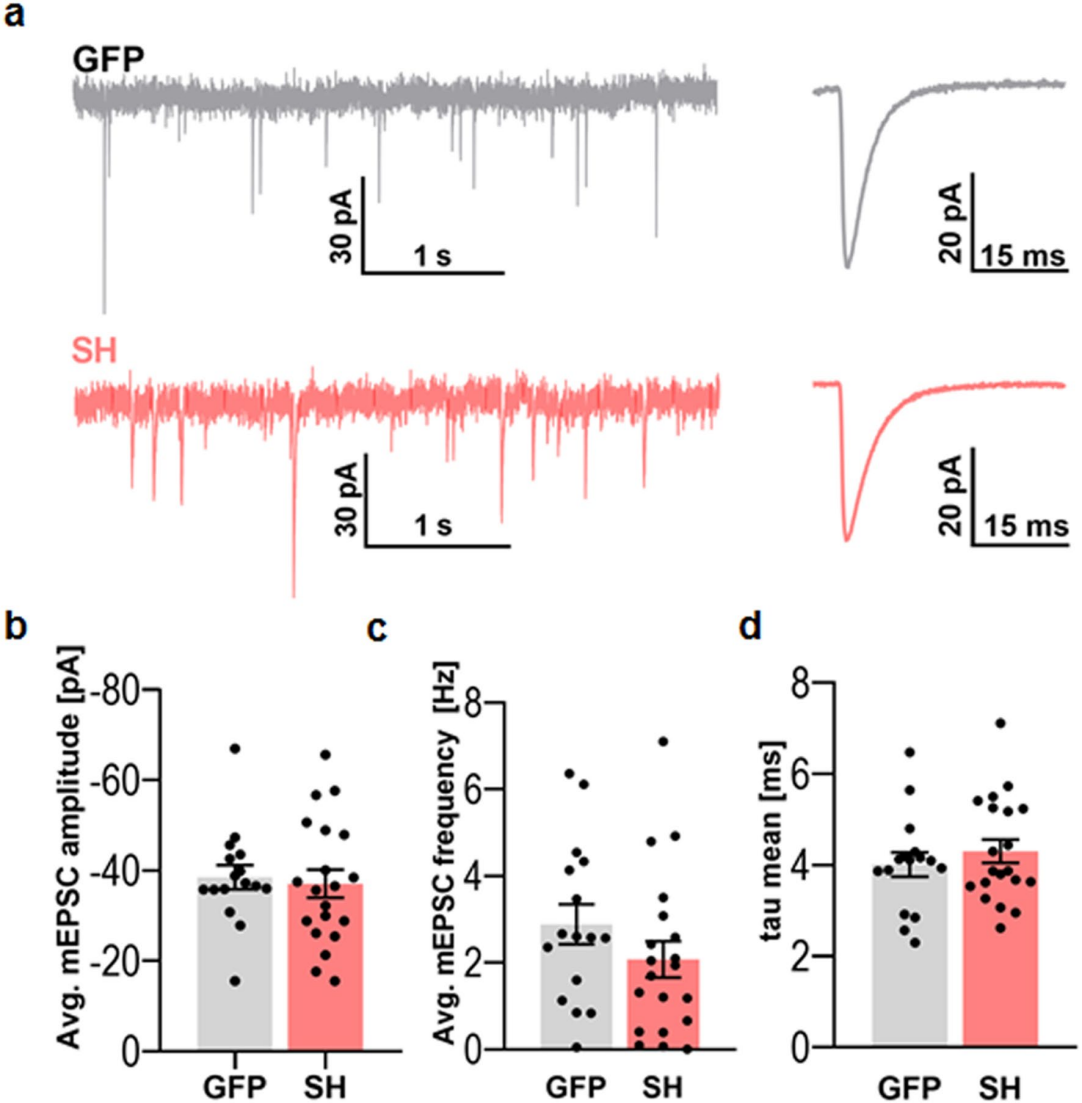
Knockdown of dystroglycan does not affect AMPA/kainate receptor glutamatergic transmission. (**a**) The left panel shows example traces of the whole-cell voltage-clamp recordings of mEPSCs in the control (GFP, gray trace) and DG knockdown (SH, red trace) neurons. The right panel shows the averaged mEPSCs. (**b–d**) Statistics for the standard parameters of AMPA/kainate mEPSCs recorded in the control neurons (GFP) and the DG knockdown neurons (SH). The manipulation of DG expression did not result in a significant alteration of mEPSC amplitude, frequency or decay time constant. The data were obtained from 16 and 20 neurons per group (N = 3 and N = 4 cultures, respectively). The results are expressed as the mean ± SEM.

It is known that the morphology of the dendritic spine correlates with the efficacy of synaptic transmission^33^. To determine the potential functional consequences of DG knockdown in hippocampal cultures, we performed recordings of synaptic transmission in individual neurons infected with lentivirus carrying either GFP or the shRNA for DG. Since the vast majority of dendritic spines are sites of excitatory synaptic transmission^34,35^, we applied the whole-cell patch-clamp technique to record miniature excitatory postsynaptic currents (mEPSCs), which are mediated by α-amino-3-hydroxy-5-methyl-4-isoxazolepropionic acid (AMPA)/kainate receptors (Fig. 8a). We found that the average amplitude and frequency of mEPSCs were similar in GFP-expressing and DG knockdown neurons (n = 16 and n = 20 cells, N = 3 and N = 4 cultures; *p* = 0.34 and *p* = 0.75, respectively, unpaired Student’s t-test, Fig. 8b and c). We then analyzed the kinetics of the recorded excitatory synaptic currents. The average rise-time kinetics of the mEPSCs (GFP: 0.67 ± 0.04 ms, SH: 0.72 ± 0.057 ms, *p* = 0.65, unpaired Student’s t-test, data not shown) and the average mEPSC deactivation constant (τ decay, *p* = 0.77, unpaired Student’s t-test, Fig. 8d) were not significantly different between the investigated groups of neurons. Thus, DG knockdown did not result in significant alteration of the efficacy or kinetics of the excitatory synaptic transmission mediated by AMPA/kainate receptors.

**Figure 9:**
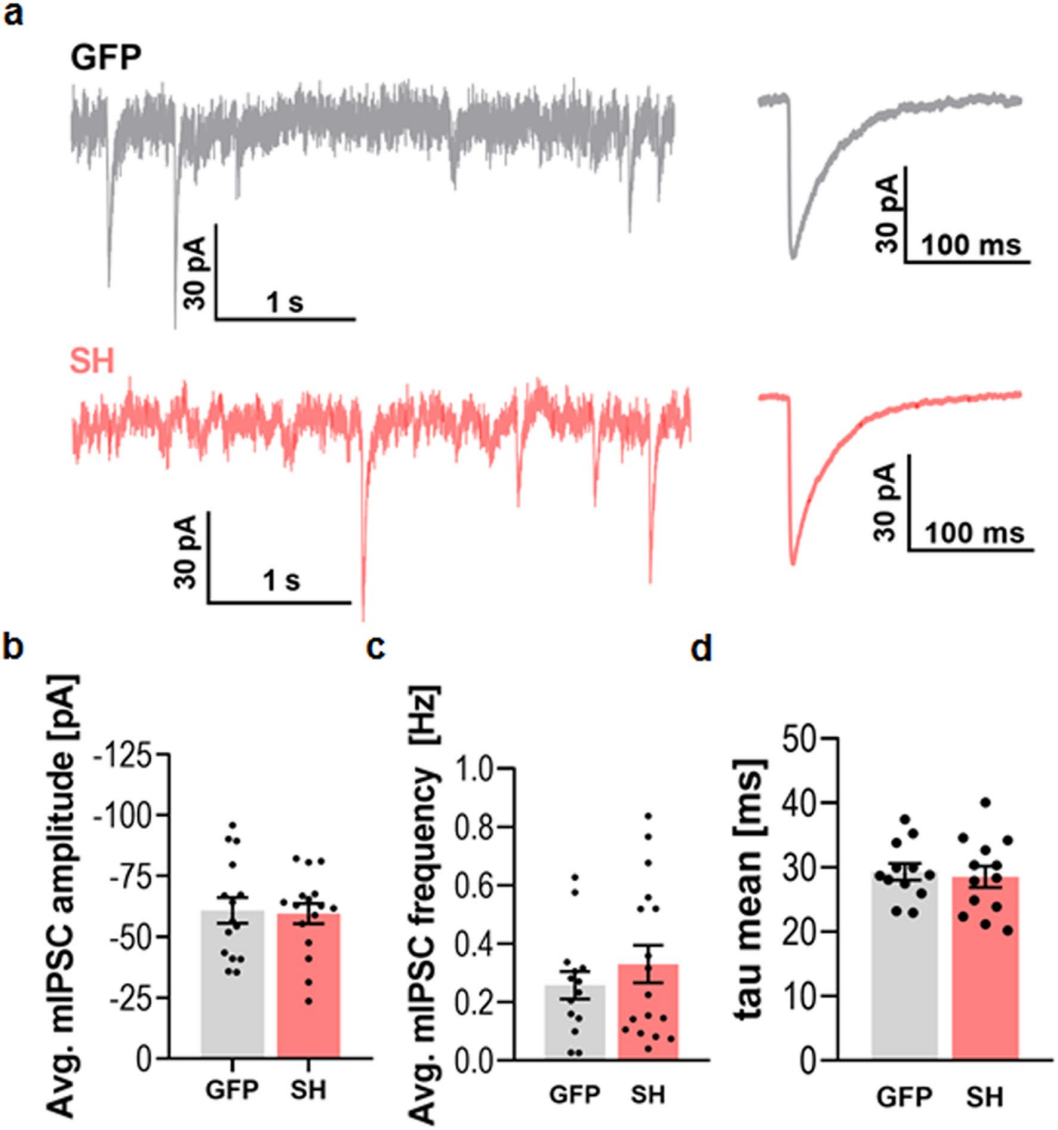
Knockdown of dystroglycan does not affect fast GABAergic synaptic transmission. (**a**) The left panel shows example traces of the whole-cell voltage-clamp recordings of mIPSCs in the control (GFP, gray trace) and DG knockdown (SH, red trace) neurons. The right panel shows the averaged mIPSCs. (**b–d**) Statistics for the mIPSC parameters recorded in the control neurons (GFP) and the DG knockdown neurons (SH). The manipulation of DG expression did not result in a significant alteration of mIPSC amplitude, frequency or decay time constant. The data were obtained from 12 and 15 neurons per group (N = 7 cultures), respectively. The results are expressed as the mean ± SEM.

DG has previously been shown to be located at GABAergic synapses but dispensable for their differentiation^11^. It has also been shown that α-DG may be involved in homeostatic synaptic plasticity at GABAergic synapses^12^. We also observed the colocalization of DG and gephyrin, the core scaffolding protein for inhibitory synapses (Supplementary Fig. S2).  Therefore, we next asked whether DG knockdown affects GABAergic synaptic transmission. To test this hypothesis, we applied the whole-cell patch-clamp technique and recorded miniature inhibitory postsynaptic currents (mIPSCs), which are mediated by GABAA receptors (Fig. 9a). We found that the average amplitude and frequency of the mIPSCs were similar in control GFP-expressing and DG knockdown neurons (n = 12 and n = 15 cells, N = 7 cultures; *p* = 0.85 and *p* = 0.34, respectively, unpaired Student’s t-test, Fig. 9b and c). Moreover, the average rise-time kinetics of the mIPSCs recorded in the GFP-expressing neurons was similar to that recorded in the DG knockdown neurons (1.42 ± 0.12 ms vs 1.44 ± 0.12 ms, respectively, *p* = 0.94, unpaired Student’s t-test, data not shown). In addition, the average mIPSC deactivation constant (τ decay) was not significantly different between the investigated groups of neurons (*p* = 0.71, unpaired Student’s t-test, Fig. 9d). These results indicate that DG knockdown did not result in alteration of the efficacy or kinetics of fast GABAergic synaptic transmission.

### Silencing of dystroglycan does not change Psd-95 expression

**Figure 10:**
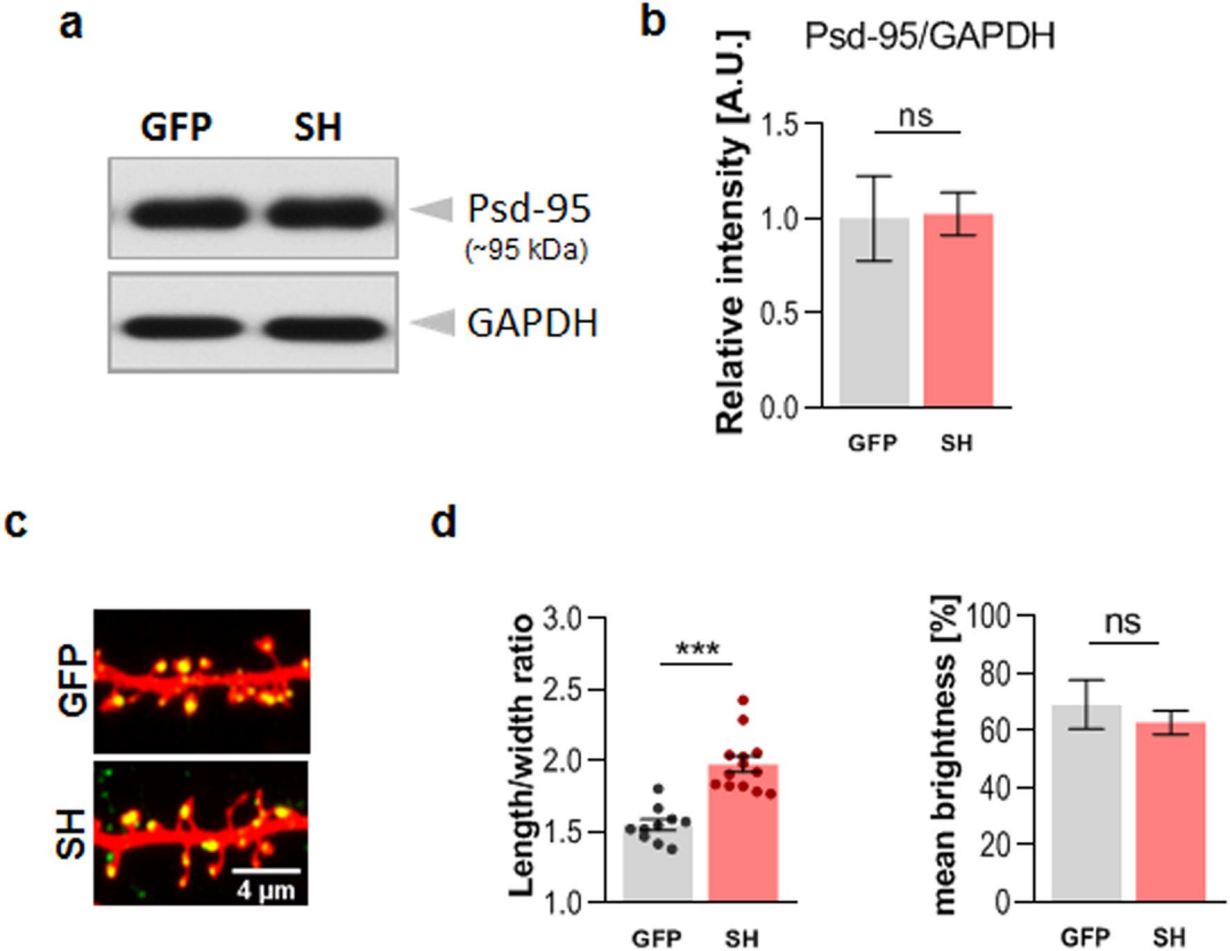
Knockdown of dystroglycan does not affect Psd-95 expression. (**a**) Representative western blot (of four independent experiments) showing the Psd-95 levels in protein extracts obtained from hippocampal cultures infected with either an empty lentivirus (GFP) or a lentivirus carrying shRNA for DG (SH). GAPDH served as a loading control. Original blots are presented in Supplementary Fig. S9. (**b**) Bar graph showing the densitometric analysis of the blots probed for Psd-95 and GAPDH. (**c**) Confocal images showing fragments of dendrites of neurons infected with the given viruses and transfected with a plasmid vector encoding RFP after immunofluorescence staining with a Psd-95-specific antibody (green). (**d**) Left: The bar plot showing the ratio of spine length to head width obtained after the morphometric analysis of the dendritic spines from (**c**). Right: The bar plot showing the mean brightness corresponding to the Psd-95 immunoreactivity within the dendritic spines from (**c**). The results are expressed as the mean ± SEM, ***p < 0.001.

Surprisingly, the results of our electrophysiological studies did not correlate with the changes in spine shape and density observed after DG knockdown. Therefore, we decided to investigate whether the downregulation of DG levels in hippocampal cultures affects the expression of Psd-95, the major scaffolding protein in the excitatory postsynaptic density and an important regulator of synaptic maturation. The western blot analysis showed that the shRNA knockdown of DG did not affect Psd-95 protein levels compared to those in the control cultures (Fig. 10a and b). Furthermore, despite the morphological changes in the dendritic spines, we did not observe any changes in Psd-95 expression within the spines, as revealed by the immunocytochemical staining of cultures infected with either the lentivirus carrying the shRNA for DG or the empty lentivirus carrying GFP (Fig. 10c and d). Thus, the knockdown of DG in primary hippocampal cultures had no effect on Psd-95 protein expression. This is not surprising considering that Psd-95 correlates with spine head size and this parameter did not change after DG silencing.

## Discussion

Dystroglycan is a ubiquitously expressed cell adhesion receptor linking the extracellular matrix to the intracellular actin cytoskeleton. Various functions have been ascribed to DG, depending on the developmental and cell-specific contexts. In the central nervous system, DG has been confirmed to be present on neuronal and glial cells. According to earlier reports, the expression of DG in central neurons is limited to the postsynaptic part of inhibitory synapses, where it associates with gephyrin and GABAA receptors^11^. Importantly, it has been shown that DG is involved in the activity-dependent pathway responsible for the homeostatic scaling up of GABAergic synaptic strength^12^. Although increased proteolytic cleavage of DG has been repeatedly confirmed both after the enhancement of neuronal activity and under pathological conditions, it is still unknown whether DG is involved in the remodeling of the synaptic structure.

The heterologous primary hippocampal cell culture we used in the present study provides a simplified system for studying the role of DG in the structural plasticity of dendritic spines, which host most of the excitatory synapses in the brain. We used a lentiviral vector to efficiently block DG expression in both neurons and astrocytes and observed significant changes in dendritic arborization as well as in dendritic spine density and morphology (Supplementary Fig. S and Fig. 2). The dendritic spines of the neurons with reduced DG expression were longer and thinner than those of the neurons expressing normal levels of DG. In addition, the spine density clearly decreased in cultures infected with the lentivirus encoding shRNA targeting DG. It should be noted that the morphology of the dendrites and spines can be affected by the density of cultures. It was shown that sparser hippocampal neurons exhibited increased spine length, lower spine density, and simpler dendritic arbors^36^. However, we strictly controlled the number of the plated cells. Moreover, we did not observe changes in the density of neurons after lentiviral infection (Supplementary Fig. S5). Interestingly, similar changes in dendritic spine morphology have been previously observed after the short-term treatment of cultured neurons with autoactivating MMP-9 (aaMMP-9), which is known to cleave DG^37^. The involvement of MMP-9 in spine formation and stabilization has been repeatedly shown^38,39^. Importantly, MMP-9 is locally translated and released from excitatory synapses in response to enhanced neuronal activity^40^. However, we would like to note that if DG is present exclusively on GABAergic synapses, its proteolytic cleavage by MMP-9, which is located only on excitatory synapses, is difficult to explain. Therefore, it does not seem possible that the proteolytic cleavage of DG located in neurons is especially important for synaptic remodeling. However, in the CNS, DG is also abundant in astroglial cells, where it assumes a unique localization at astrocytic endfeet, which cover blood vessels and synapses^41^. The brain-specific deletion of DG during development resulted in brain malformations and the disorganization of astroglial endfeet structures^9^. Since astrocytic endfeet play a critical role in the maintenance of the regulated flow of molecules in the brain, disturbances in DG expression can affect the progression of brain pathophysiology. One example of such a phenomenon was provided by a study by Gondo and colleagues^42^, in which the authors showed that the continuous epileptic activation of neurons in acute brain slices from mice leads to a decrease in DG expression and associated dysfunction of the astrocytic endfeet. Notably, the observed reduction in DG was completely abolished by the preincubation of brain slices with specific MMP inhibitors. Although MMP-9 was detected primarily in neuronal cells in the rat hippocampus, its presence in a fraction of GFAP-positive astrocytes has been shown^43^. It has also been suggested that MMP-9 may act preferentially at the level of glial processes, where its activity is not inhibited by the endogenous inhibitor TIMP-1 (tissue inhibitor of metalloproteinase-1)^44^. Although there is no evidence that DG is locally cleaved by MMP-9 in astrocytic endfeet, the colocalization of these proteins suggests that this process may occur. However, it is known that the degradation of cell adhesion proteins modifies cell–cell and cell-extracellular matrix interactions, leading to structural and functional changes in synapses^45^.

In the present study we observed that the knockdown of DG causes changes in astroglial cell morphology (Fig. 7). Astrocytes from the DG-deficient cultures showed decreased expression of pEzrin, a key component of perisynaptic processes^32^. We further found that DG silencing was accompanied by a decrease in AQP4 expression, which is predominantly expressed in astrocyte endfeet (Fig. 6a). It has been previously demonstrated that the AQP4 protein regulates the motility of these processes and can thus influence the physiological interplay between astrocytes and neurons^29^. Our results are in agreement with previous reports showing that AQP4 clustering at the cell surface is significantly reduced in primary astrocyte cultures transfected with siRNA targeting the DG gene^30^. The same study also provided evidence that the localization of AQP4 at the endfeet of astroglial processes is crucially dependent on the laminin-binding properties of DG. These data were further supported by experiments performed on mouse astrocyte cultures derived from neurospheres^46^. Our results may also indicate that laminin has a role in the mechanism that regulates AQP4 expression in hippocampal astrocytes. Indeed, the western blot analysis and immunofluorescence staining revealed the reduced expression of laminin in DG-deficient cultures (Fig. 6b). These results may indicate that the reduction of DG expression disrupts the interactions between cells and the extracellular matrix, which may contribute to changes in the structure of the dendritic spines. This assumption is reasonable as several reports indicate that laminin modulates synaptic differentiation (reviewed in:^47^.

We further found that DG knockdown caused the formation of filopodial projections growing from the heads of mature dendritic spines, the so-called spine head protrusions (SHPs; Fig. 2e). Previous studies by Verbich and colleagues^48^ have shown that perisynaptic astrocyte remodeling and glutamate uptake are involved in the formation of SHPs in hippocampal slices after incubation with tetrodotoxin (TTX) and glutamate iontophoresis. Importantly, using time-lapse confocal microscopy, they revealed that the volume overlap between spines and astrocytic processes decreased during the formation of SHPs. It was also reported that SHP-expressing spines are characterized by the increased expression of the AMPA receptor GluA1 and GluA2 subunits, whose recruitment is closely related to MMP-9 activity^49^. Thus, dynamic changes of both spines and astrocytes can rapidly modify synaptic connectivity. We did not observe that DG silencing affected Psd-95 levels. Moreover, the expression of Psd-95 within the spines remained unchanged and did not correlate with the altered length to head width ratio (Fig. 10). However, since the knockdown of DG did not lead to changes in the spine head width (Fig. 2c), it is reasonable that there were no changes in Psd-95 immunoreactivity. It has been repeatedly shown that spine volume and Psd-95 expression are linearly related^50,51^.

Surprisingly, the aberrant structural remodeling after the knockdown of DG did not have an impact on the functional properties of AMPAR-mediated fast synaptic transmission (Fig. 8). Significant spine elongation was previously shown to result in the prolongation of the kinetics and the magnitude of synaptic transmission^52,53^. However, such changes in mEPSCs were not observed in our study. A possible explanation might be that the mEPSCs were recorded from a random subset of the dendritic spines, while in the structural analysis, all dendritic spines per dendrite were analyzed. Thus, direct translation between the obtained structural and functional data may not be possible. Moreover, Psd-95 expression was previously shown to positively correlate with the magnitude of excitatory synaptic transmission^54^. In our study, this factor remained unchanged following DG knockdown and thus supported the observed electrophysiological data. Additionally, the spine head width was not altered in the DG knockdown neurons (Fig. 2c). In summary, it appears that DG knockdown in primary hippocampal cultures more significantly affects the structure of dendritic spines and certain structural features of astrocytes than the function of excitatory synapses. In addition, we did not observe any significant changes in GABAergic transmission despite the high abundance of both DG subunits in the GABAergic synapses (Fig. 9). In this respect, our data confirm the results of a previous study in which knockdown of α-DG did not result in altered phasic GABAergic transmission^12^.

Taken together, our data indicate that in mature hippocampal neurons, DG is more likely to regulate structural plasticity than acute synaptic function. Interestingly, reduced spine density caused by DG shRNA was partially rescued by overexpression of DG in astroglial cells (Fig. 4). In this regard, the DG expressed in astrocytes may play a pivotal role in synapse stabilization and compensatory mechanisms; however, further experimental insights will be needed to directly reveal this phenomenon.

## Materials and methods

### Primary hippocampal cultures

The animal procedures and protocols were carried out following the guidelines established and approved by the Polish Ethical Committee on Animal Research. Dissociated hippocampal cultures were prepared from postnatal day 0 (P0) Wistar rats as described previously^55^. For the morphometric analysis of dendritic spines, the electrophysiological studies, and the immunocytochemical staining, cells were plated at a density of 75,000 cells per 13-mm-diameter coverslip (Assistant, Germany) coated with 50 µg/ml poly-D-lysine (Sigma, Saint Louis, USA). For the western blot experiments, cells were plated on 6-well poly-D-lysine-coated tissue culture plates at a density of 120,000 cells per well. The cultures were kept at 37 °C in 5% CO2 in a humidified incubator. The experiments were performed at 15–21 day in vitro (DIV).

### Overexpression and silencing of dystroglycan

A plasmid vector encoding the DG sequence (WT) and a lentiviral vector carrying shRNA for DG (SH) were prepared as previously described^26^. Briefly, the lentiviral vector co-expresses shRNA sequence for DG under the U6 promoter and GFP under the synapsin promoter. The U6 promoter enables the expression of shRNA in any type of cell (both neurons and astrocytes), while the expression of GFP under the synapsin promoter enables specific visualization of neurons. The cultures were infected with the given lentivirus on 9 DIV. We selected this time point because intensive dendritogenesis in primary hippocampal neurons occurs during the first week in vitro and our previous studies have confirmed the role of DG in this process^26^. To visualize the dendritic spines of neurons with silenced DG expression, the cultures were first infected with virus carrying shRNA targeting DG (on 9 DIV) and then transfected with a plasmid containing the red fluorescent protein (RFP) sequence (on 12 DIV) using the Lipofectamine 2000 Reagent (ThermoFisher Scientific, Waltham, MA, USA) according to the manufacturer’s protocol. To study the effect of DG overexpression on dendritic spine morphology, the cultures were cotransfected with the WT DG plasmid and a plasmid encoding RFP under the synapsin promoter on the 9th DIV.

To generate a plasmid vector encoding the DG shRNA-resistant forms (rescue) cDNA of DG was subcloned into pTRIP vector (pTrip-GFP). The pTrip-GFP plasmid was cleaved with BstBI and NheI enzymes, and the OE DG plasmid^26^ was cleaved with ClaI and NheI enzymes, which resulted in the generation of plasmid pTrip-Syn-DAG. A silent point mutation was then introduced to create a shRNA-resistant version of the DG (sequence TGTCGGCACCTCCAATTT has been changed to TGCCGACATCTTCAGTTC) (pTrip-Syn-DAG-rescue). In the next step, the HA tag was added before the STOP codon to create pTrip-Syn-DAG-rescue-HA plasmid. Then, GFaABC1D promoter was subcloned from pZac21gfaABC1D_MCS plasmid into pTrip-Syn-DAG-rescue-HA plasmid to create pTrip-GFAP-DAG-rescue-HA plasmid. PZac21gfaABC1D_MCS plasmid was cleaved with BglII and NheI and pTrip-Syn-DAG-rescue-HA plasmid was cleaved with MluI and NheI. The cloning was performed by the GenScript company (https://www.genscript.com/).

### Morphometric analysis of dendritic spines

The images of neuronal dendrites were acquired using a confocal microscope (Zeiss LSM 780) with a PL Apo 40 × /1.25 NA oil immersion objective with 488 nm and 561 nm diode-pumped solid-state lasers at 10% transmission at a pixel count of 1024 × 1024. A series of z-stacks were collected for each cell with a 0.4 μm step size with additional digital zoom resulting in a lateral resolution of 0.07 μm per pixel size. The morphometric analysis of dendritic spines was performed semiautomatically using the SpineMagick software (patent no. WO/2013/021,001) as we previously described^37^. Spines belonging to secondary and tertiary dendrites of pyramidal neurons were analyzed to reduce the possible differences in spine morphology caused by their location on dendrites with different ranks. We used a scale-free parameter of relative changes in the spine length-to-head width ratio, which reflects spine shape. The spine length was determined by measuring the curvilinear length along with a fitted virtual skeleton of the spine. The fitting procedure was performed by looking for a curve along which integrated fluorescence was at a maximum. We did not take into account the width of the spine neck due to the limitation of the microscopic technique used. The head width was defined as the diameter of the largest spine section excluding the bottom part of the spine (1/3 of the spine length adjacent to the dendrite). The dendritic spines of at least three primary hippocampal cultures were analyzed.

### Immunocytochemistry and image analysis

Immunofluorescence staining was carried out as we previously described^26^, with minor modifications. The hippocampal cultures were fixed in a mixture of 4% (wt/vol) paraformaldehyde (PFA)/4% (wt/vol) sucrose/PBS for 6 min at room temperature and permeabilized with 0.1% Triton X-100 in PBS (PBST) for 5 min. After nonspecific binding sites were blocked with 5% bovine serum albumin (BSA)/PBST for 1 h at room temperature, the cells were incubated with the primary antibodies diluted in blocking buffer at 4 °C overnight. The following primary antibodies were used: mouse anti-β-DG (1:100; sc-33702, Santa Cruz Biotechnology, Dallas, TX, USA), rabbit anti-laminin (1:200; L9393, Sigma-Aldrich, Saint Louis, USA), mouse anti-Psd-95 (1:500; MABN68, Millipore, Darmstadt, Germany), chicken anti-MAP2 (1:5000; ab5392, Abcam, Cambridge, UK), mouse anti-GFAP (1:300; G3893, Sigma-Aldrich, Saint Louis, USA), and rabbit anti-pERM (1:200; 3726, Cell Signaling Technology, Danvers, MA, USA).. After the cells were washed with PBS, secondary antibodies conjugated with Alexa Fluor 555 or Alexa Fluor 647 (ThermoFisher Scientific, Waltham, MA, USA) were applied for 1 h at room temperature. Following washing with PBS, the coverslips were mounted in an anti-quenching medium (Fluoromount G, Southern Biotechnology Associates, Biozol, Eching, Germany) and subjected to imaging analysis. The images were acquired under 555 nm and 650 nm fluorescent illumination using a Zeiss LSM 780 confocal microscope (63 × objective, 1.4 NA).

### Electrophysiological recordings

Whole-cell patch-clamp recordings in voltage-clamp mode were performed in hippocampal cultures at 15–17 or 20–21 DIV using borosilicate patch pipettes (2.5–4.5 MΩ) filled with one of the intracellular solutions described below. The external solution contained 125 mM NaCl, 5 mM KCl, 2 mM CaCl2, 1 mM MgCl2, 20 mM glucose, and 10 mM HEPES (pH 7.3, adjusted with NaOH). The miniature excitatory postsynaptic currents (mEPSCs) were recorded at a holding potential of − 60 mV after bath application of 10 μM gabazine and 1 μM tetrodotoxin (TTX) with an internal solution containing 116 mM potassium gluconate, 6 mM KCl, 2 mM NaCl, 0.5 mM EGTA, 20 mM HEPES, 4 mM MgATP, 0.3 mM NaGTP, and 10 mM Na2 phosphocreatine (pH 7.3). After the end of recording, 20 μM AMPA/kainate receptor antagonist 6,7-dinitroquinoxaline-2,3-dione (DNQX) was used to confirm the origin of the recorded mEPSCs and the lack of GABAergic currents.

The miniature inhibitory postsynaptic currents (mIPSCs) were recorded at a holding potential of − 60 mV in the presence of 1 μm TTX and 20 μM DNQX with an internal solution containing 140 mM KCl, 1 mM MgCl2, 0.5 mM EGTA, 10 mM HEPES, and 4 mM MgATP (pH 7.3). After the end of recording, 10 μM gabazine was used to confirm the origin of the recorded mIPSCs. All of the chemicals were purchased from Sigma-Aldrich (Saint Louis, USA), with the exception of TTX (Latoxan, Valence, France). The experiments were performed at room temperature (23–24 °C), and the drugs were bath-applied in the extracellular solution perfused at 3 mL/min.

All recorded signals were low-pass filtered at 10 kHz using the eight-pole Bessel filter built into the Multiclamp 700B patch-clamp amplifier (Molecular Devices, LLC, San Jose, CA, USA), digitized at 20 kHz (Digidata 1550B, Molecular Devices), and acquired with the pClamp 11 software (Molecular Devices). All the data were subsequently analyzed using Clampfit (Molecular Devices) and Prism 8 (GraphPad Software, CA, USA) as previously described^36^. The series resistance (Rs) was estimated from the response to a hyperpolarizing voltage step (− 5 mV)^56^. The recordings in which series resistance was > 20 MΩ were rejected.

### Western blot

Western blot analysis was performed as we previously described^15^, with slight modifications. The hippocampal cells were lysed in RIPA buffer (Sigma Aldrich, Saint Louis, USA) that contained 150 mM NaCl, 1.0% IGEPAL CA-630, 0.5% sodium deoxycholate, 0.1% SDS, and 50 mM Tris, pH 8.0. The protein lysates (30 μg) were then separated by SDS-PAGE and transferred to polyvinylidene difluoride membranes (Immobilon-P, Millipore, Darmstadt, Germany). The membranes were then blocked with 10% nonfat milk in Tris-buffered saline with 0.1% Tween 20 (TBST). After blocking, the membranes were incubated overnight at 4 °C with the following primary antibodies diluted in 5% nonfat milk in TBST: mouse anti-β-DG (1:500; sc-33702, Santa Cruz Biotechnology, Dallas, TX, USA), rabbit anti-α-DG (1:500; ab199768, Abcam, Cambridge, UK), mouse anti-AQP4 (1:500; ab81355, Abcam, Cambridge, UK), mouse anti-GFAP (1:10,000; G3893, Sigma-Aldrich, Saint Louis, USA), rabbit anti-laminin (1:1000; L9393, Sigma-Aldrich, Saint Louis, USA), mouse anti-Psd-95 (1:5000; MABN68, Millipore, Darmstadt, Germany), and mouse anti-GAPDH (1:15,000; MAB374, Millipore, Darmstadt, Germany). The blots were washed three times with TBST then incubated for 1 h with peroxidase- conjugated secondary antibody diluted 1:3000 in TBST containing 5% nonfat milk. After washing, the bands were detected using the ECL Prime chemiluminescent detection system (GE Healthcare).

### Statistical analysis

The statistical analyses were performed using the GraphPad Prism 8 software (GraphPad Software, San Diego, CA, USA). The quantitative analysis of the western blots was performed by the sum of replicates^57^. The unpaired two-tailed Student’s t-test was used to determine significant differences between the experimental groups. In the case of unequal variance, the Welch correction was applied. If the normal distribution criterion was not achieved, the nonparametric Mann–Whitney test was used. A *p* value of < 0.05 was considered statistically significant. The data are represented as the mean value ± SEM.

## Supplementary Information

Supplementary Information.
