# Supplementary material for: The cell adhesion protein dystroglycan affects the structural remodeling of dendritic spines
Source: Sci Rep. 2022 Feb 15;12:2506. doi: 10.1038/s41598-022-06462-7 (PMC8847666; doi:10.1038/s41598-022-06462-7)
Supplement: Supplementary file 1 — Supplementary Information. [file 41598_2022_6462_MOESM1_ESM.pdf]

Supplementary Figures

Figure S1. Mature primary hippocampal cultures contain neurons and astrocytes. Confocal images showing 20-day-old hippocampal culture after immunostaining for MAP2 (green) and GFAP (red). Cell nuclei were stained with DAPI (blue).

Figure S2. Dystroglycan colocalizes with gephyrin in hippocampal neurons. Upper panel: Confocal images of cultures immunostained with anti-β-DG (red), anti-gephyrin (green), and anti-MAP2 (blue). Lower panel: Merged image and enlarged image of region enclosed in rectangle showing the colocalization of β-DG and gephyrin along the dendrites.

Figure S3. Dystroglycan controls dendritic arborization. Hippocampal cultures were infected on the 9th day after seeding with a lentivirus carrying shRNA for DG (SH) or with an empty lentivirus carrying only GFP (GFP) and additionally transfected with RFP-encoding vector. After 11 days, the cultures were subjected to morphometric analysis. **(a)** The bar plot showing the total dendritic length. **(b)** Sholl analysis of neurons treated as indicated (N=14 neurons per group). The data are expressed as mean ± SEM. $***p < 0.001$; $*p < 0.05$.

Figure S4. Knockdown of dystroglycan does not affect the levels of GFAP. Western blots showing the β-DG and GFAP protein levels in the lysates from hippocampal cell cultures infected with a virus carrying shRNA against DG (SH) or a virus carrying the GFP gene (GFP). GAPDH was used as a loading control. Original blots are presented in Supplementary Fig. S10.

Figure S5. **Knockdown of dystroglycan does not influence the density of cultures.** Confocal images showing hippocampal cultures after infection with a virus carrying shRNA against DG (SH) or an empty virus (GFP) and immunofluorescence staining with anti-GFAP antibody (red).

Figure S6. Images of original western blots. Ponceau S staining of the membranes before immunoblotting and original uncropped images of the X-ray films used to capture the chemiluminescent signal corresponding to Fig. 1 (α-DG and β-DG). The different exposure times are shown. Red boxes mark the bands of interest. N.I. – non-infected.

Figure S7. Images of original western blots. Ponceau S staining of the membranes before immunoblotting and original uncropped images of the X-ray films used to capture the chemiluminescent signal corresponding to Fig. 6a (AQP4). The different exposure times are shown. The blots were cut, since the silencing efficiency of dystroglycan was simultaneously tested. Red boxes mark the bands of interest. N.I. – non-infected.

Figure S8. Images of original western blots. Ponceau S staining of the membranes before immunoblotting and original uncropped images of the X-ray films used to capture the chemiluminescent signal corresponding to Fig. 6b (laminin). The different exposure times are shown. The blots were cut, since the silencing efficiency of dystroglycan was simultaneously tested. Red boxes mark the bands of interest.

Figure S9. Images of original western blots. Ponceau S staining of the membranes before immunoblotting and original uncropped images of the X-ray films used to capture the chemiluminescent signal corresponding to Fig. 10a (Psd-95). The different exposure times are shown. The blots were cut, since the silencing efficiency of dystroglycan was simultaneously tested. Red boxes mark the bands of interest.

Figure S10. Images of original western blots. Ponceau S staining of the membranes before immunoblotting and original uncropped images of the X-ray films used to capture the chemiluminescent signal corresponding to Fig. S4 (β-DG and GFAP). The different exposure times are shown. Red boxes mark the bands of interest.

Figure S1

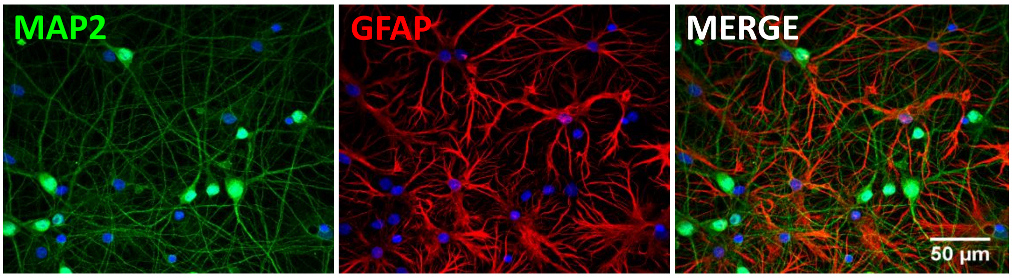

Figure S2

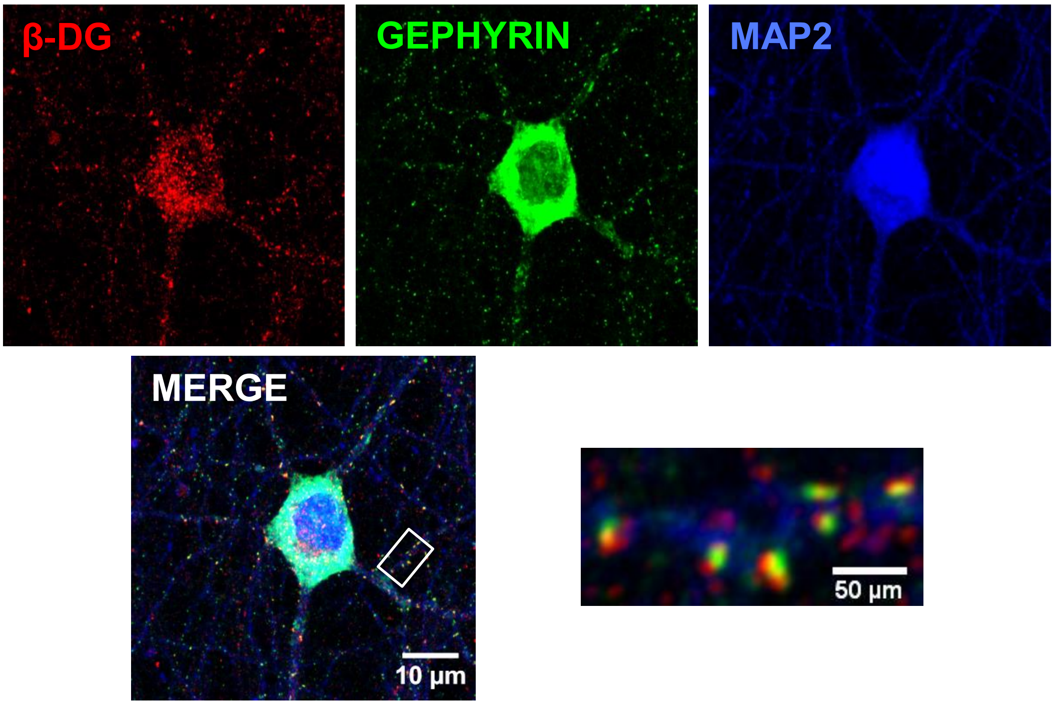

Figure S3

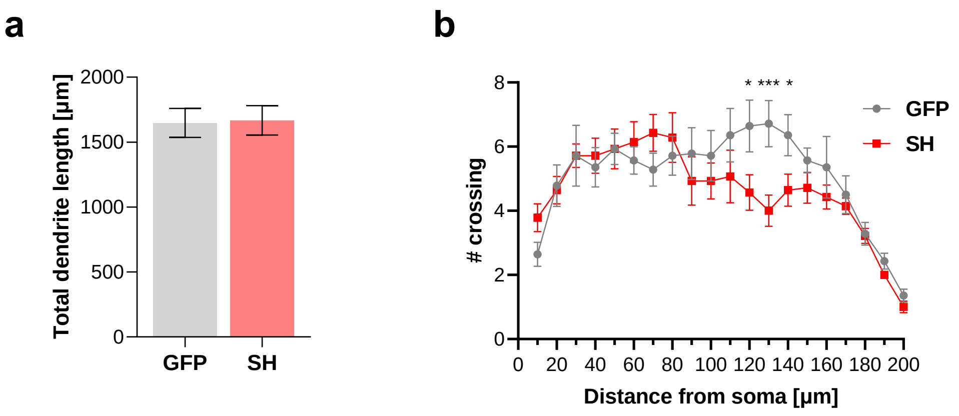
a
Total dendrite length [µm]
2000
1500
1000
500
0
GFP
SH
b
crossing
8
6
4
2
0
* *** *
GFP
SH
0 20 40 60 80 100 120 140 160 180 200
Distance from soma [µm]

Figure S4

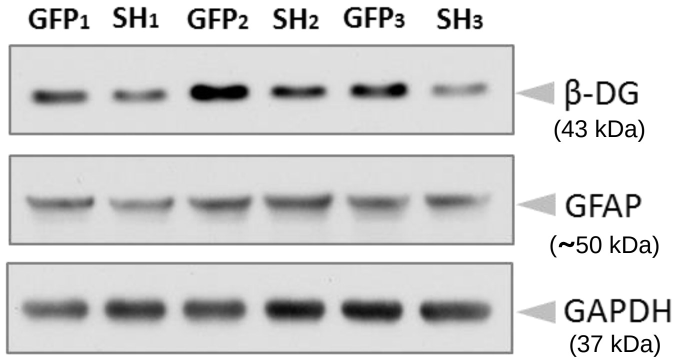

Figure S5

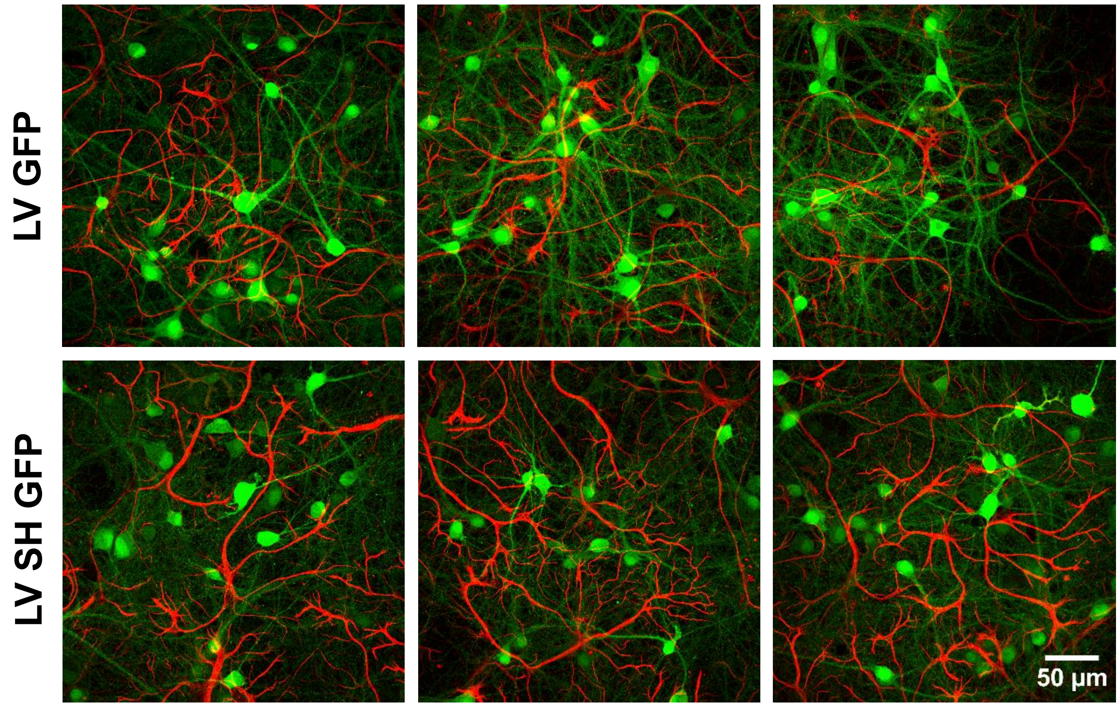

Figure S6

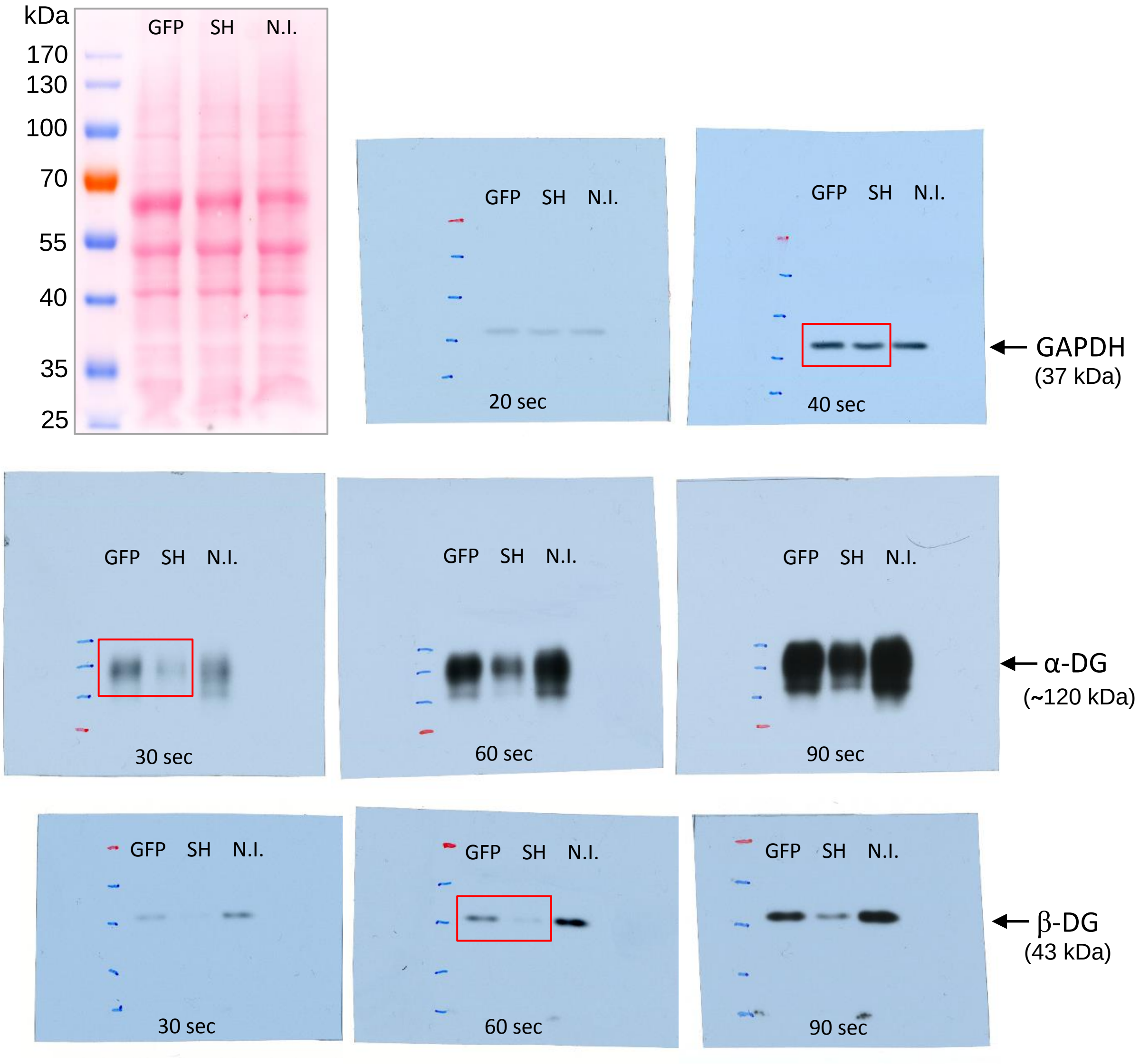
kDa
170
130
100
70
55
40
35
25
GFP SH N.I.
20 sec
40 sec
GAPDH
(37 kDa)
30 sec
60 sec
90 sec
α-DG
(~120 kDa)
30 sec
60 sec
90 sec
β-DG
(43 kDa)

Figure S7

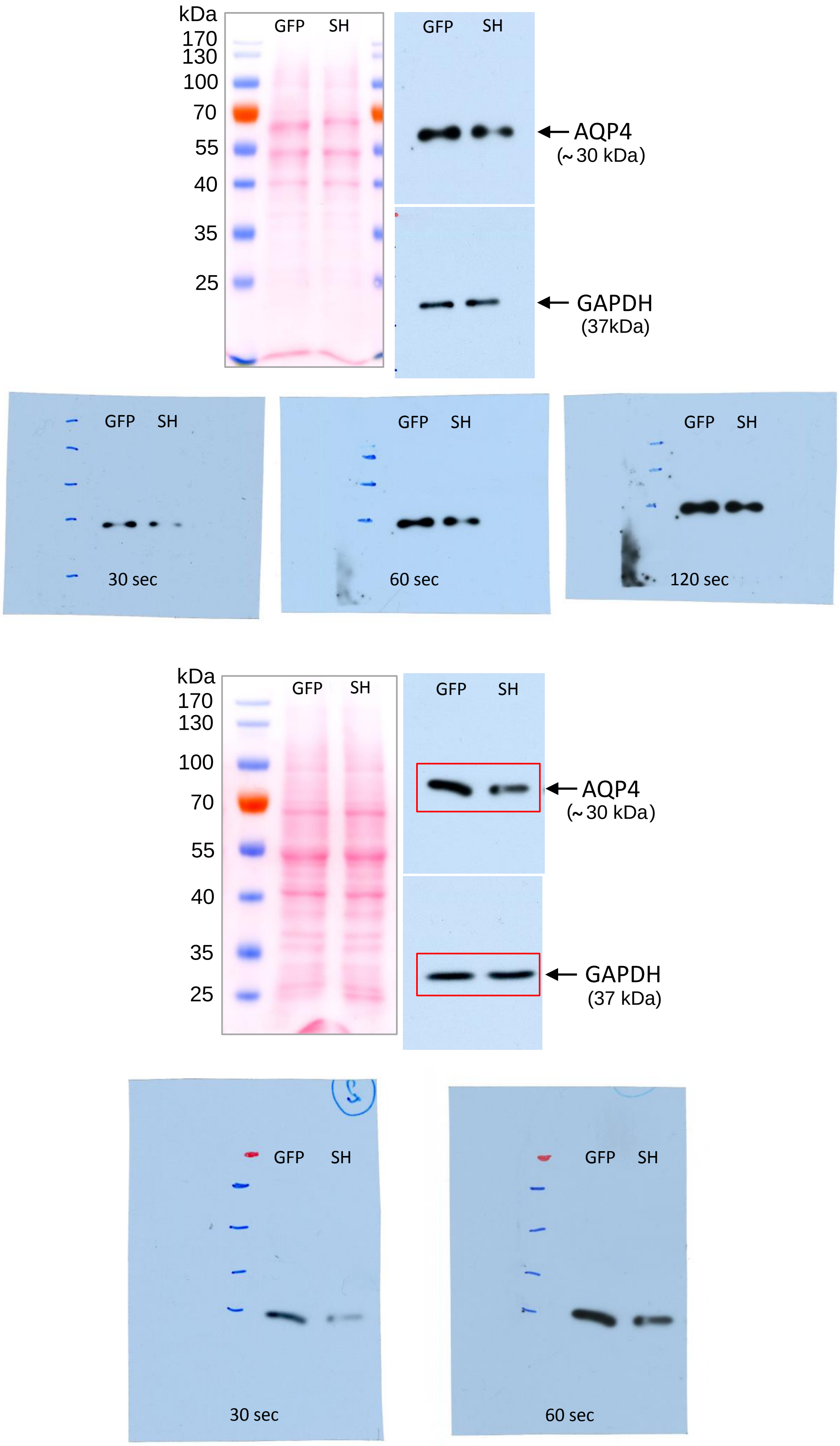
kDa
170
130
100
70
55
40
35
25
GFP SH
GFP SH
AQP4
(~30 kDa)
GAPDH
(37kDa)
GFP SH
30 sec
GFP SH
60 sec
GFP SH
120 sec
kDa
170
130
100
70
55
40
35
25
GFP SH
GFP SH
AQP4
(~30 kDa)
GAPDH
(37 kDa)
GFP SH
30 sec
GFP SH
60 sec

Figure S7

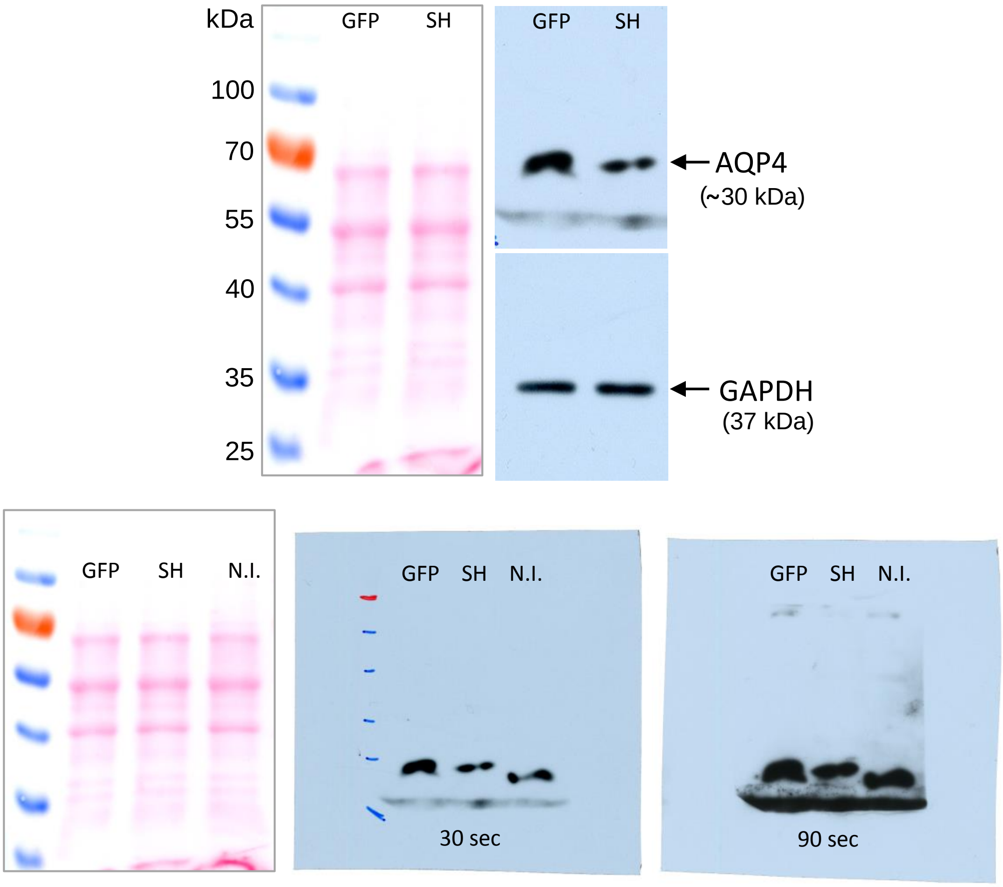

kDa
GFP
SH
100
70
55
40
35
25
GFP
SH
AQP4
(~30 kDa)
GAPDH
(37 kDa)
GFP
SH
N.I.
GFP
SH
N.I.
30 sec
GFP
SH
N.I.
90 sec

Figure S8

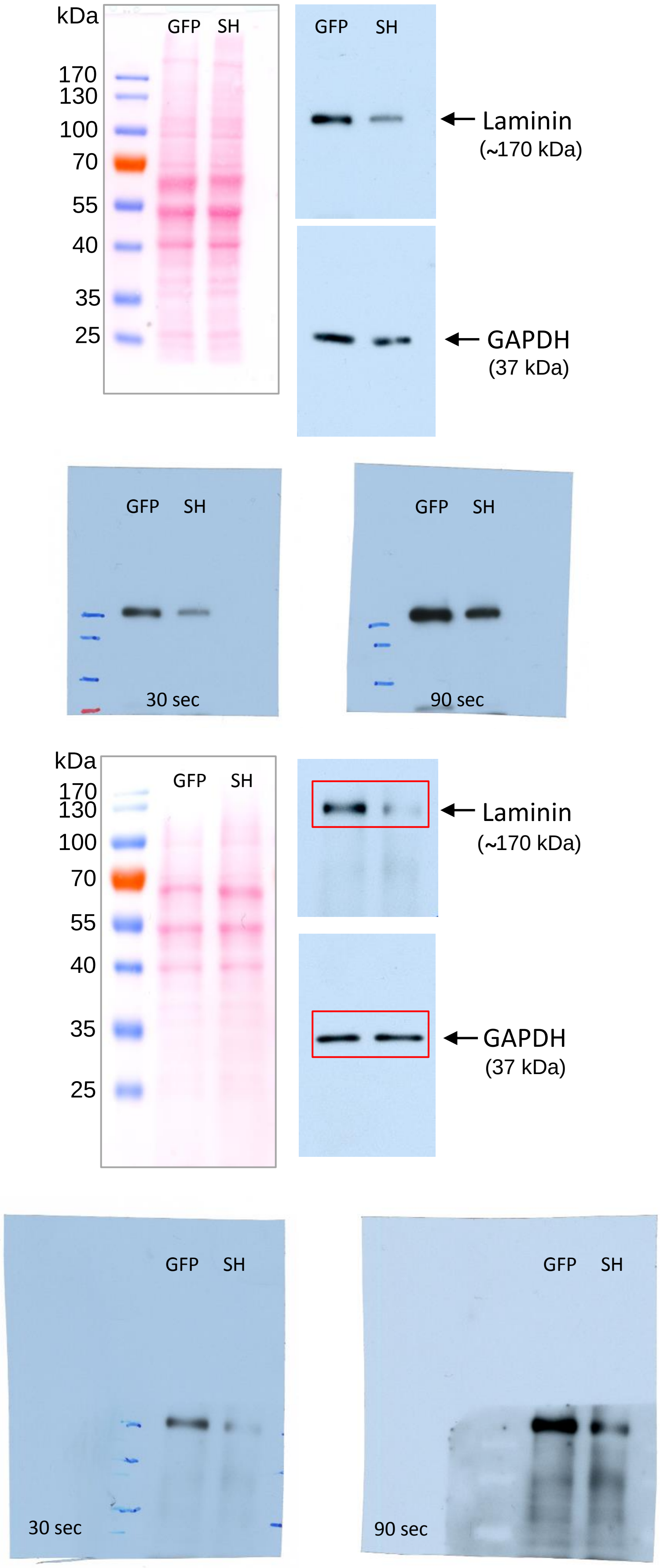
kDa
170
130
100
70
55
40
35
25
GFP SH
GFP SH
Laminin
(~170 kDa)
GAPDH
(37 kDa)
GFP SH
30 sec
GFP SH
90 sec
kDa
170
130
100
70
55
40
35
25
GFP SH
Laminin
(~170 kDa)
GAPDH
(37 kDa)
GFP SH
30 sec
GFP SH
90 sec

Figure S8

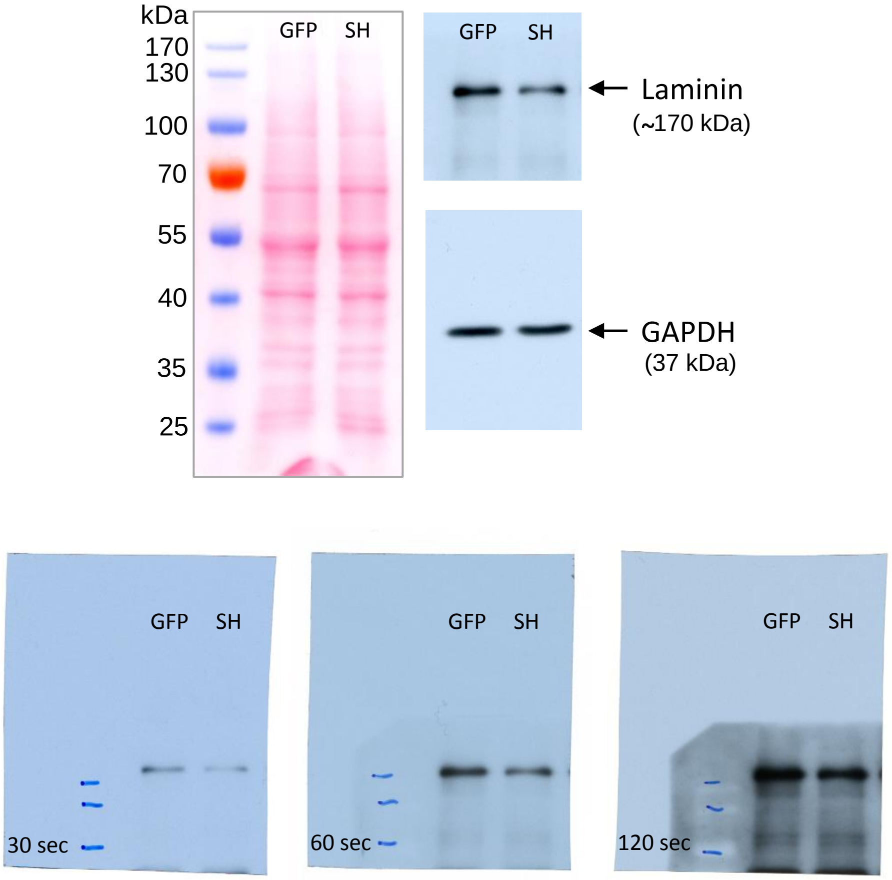
kDa
170
130
100
70
55
40
35
25
GFP SH
GFP SH
Laminin
(~170 kDa)
GAPDH
(37 kDa)
GFP SH
30 sec
GFP SH
60 sec
GFP SH
120 sec

Figure S9

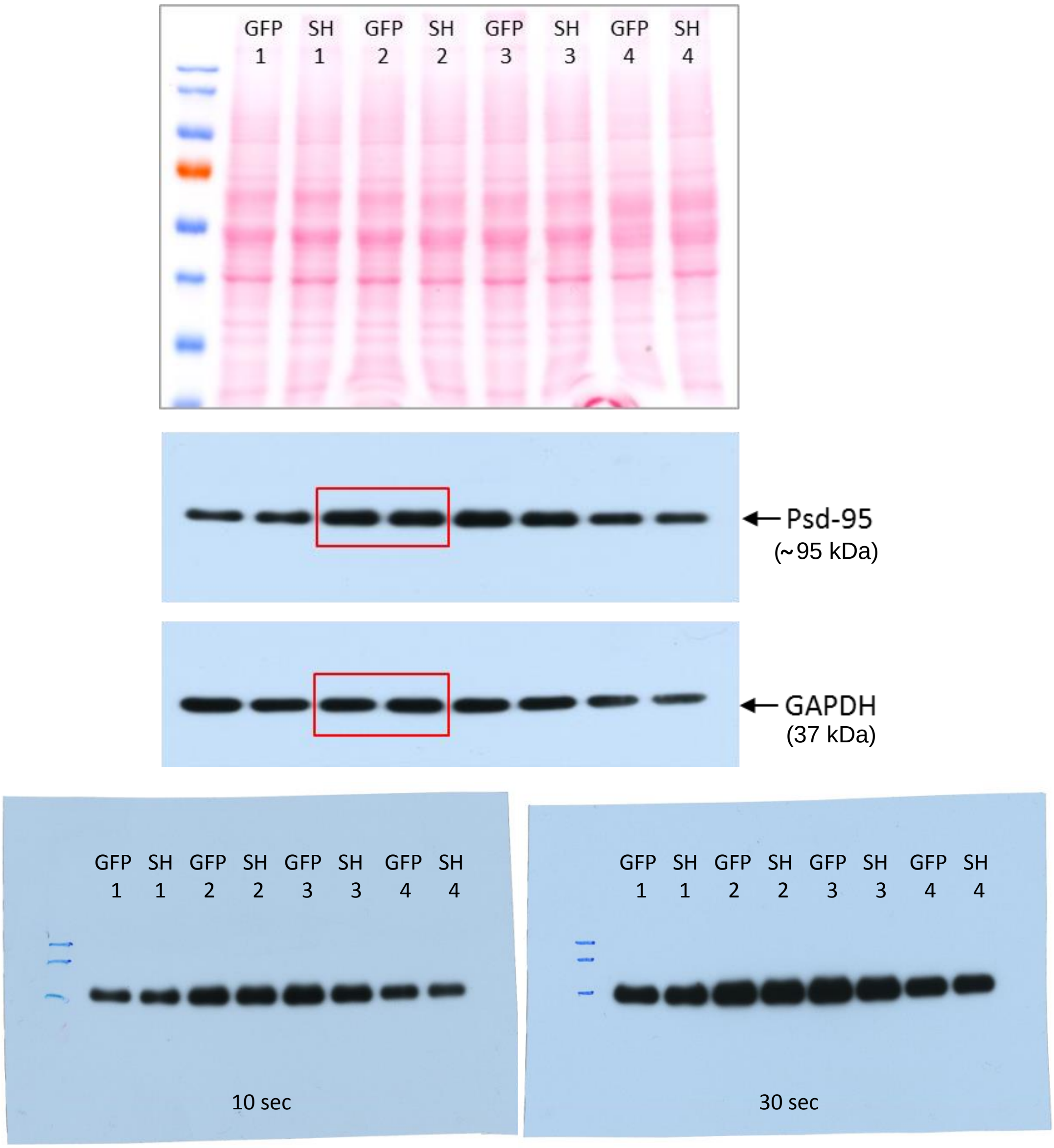
GFP 1
SH 1
GFP 2
SH 2
GFP 3
SH 3
GFP 4
SH 4
Psd-95
(~95 kDa)
GAPDH
(37 kDa)
10 sec
30 sec

Figure S10

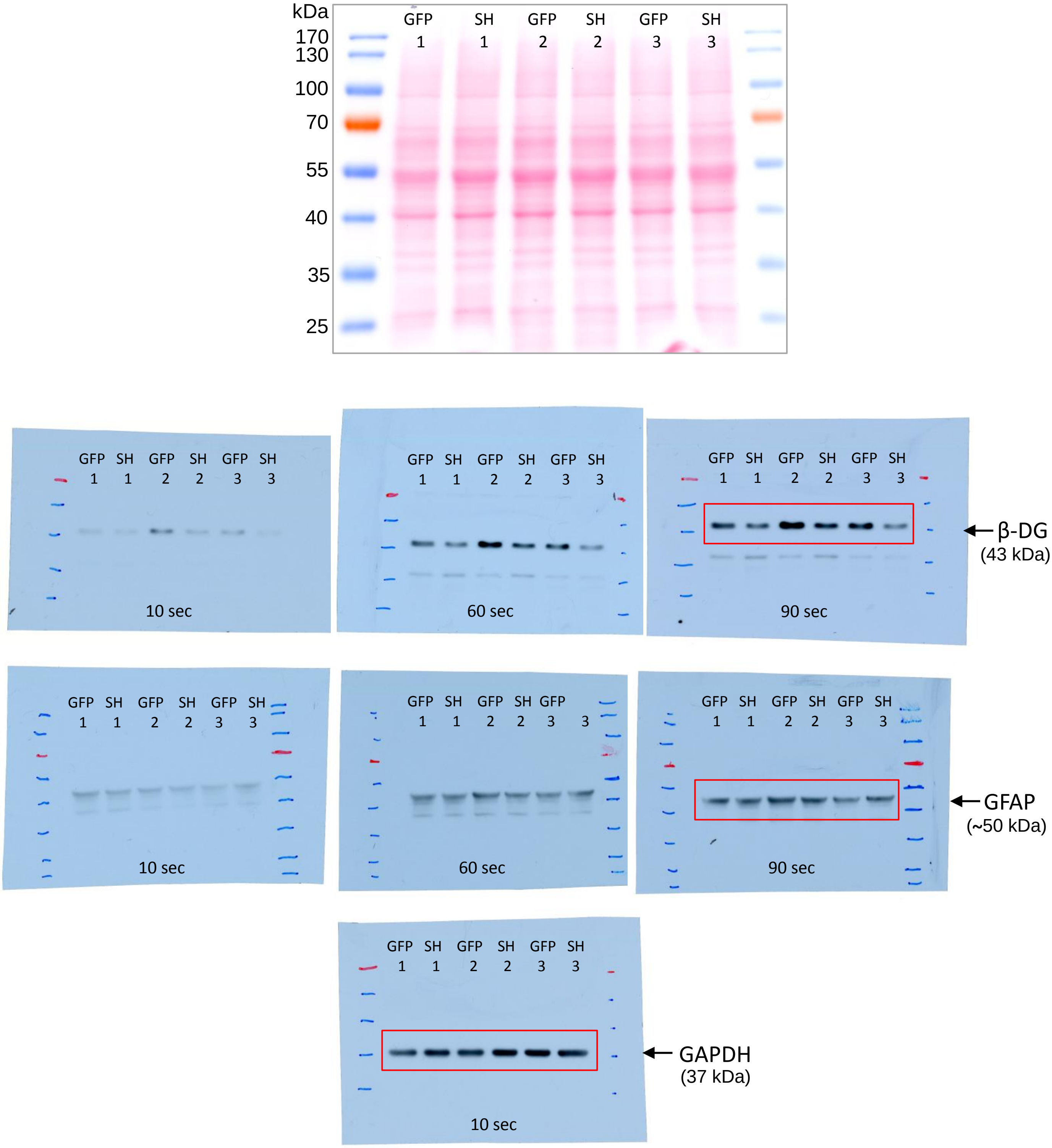

kDa
170
130
100
70
55
40
35
25
GFP 1
SH 1
GFP 2
SH 2
GFP 3
SH 3
10 sec
60 sec
90 sec
β-DG
(43 kDa)
GFAP
(~50 kDa)
GAPDH
(37 kDa)
